# A telescopic microscope equipped with a quanta image sensor for live-cell bioluminescence imaging

**DOI:** 10.1038/s41592-025-02694-3

**Published:** 2025-05-29

**Authors:** Ruyu Ma, Luciano M. Santino, Tomáš Chobola, Niklas Armbrust, Julian Geilenkeuser, Sapthagiri Sukumaran, Zhizi Jing, Anastasia Levkina, Korneel Ridderbeek, Tingying Peng, Dong-Jiunn Jeffery Truong, Sebastian Doll, Gil Gregor Westmeyer, Jian Cui

**Affiliations:** 1 Helmholtz Pioneer Campus, Helmholtz Munich, Neuherberg, Germany; 2 Helmholtz AI, Helmholtz Munich, Neuherberg, Germany; 3 TUM School of Computation, Information and Technology (CIT), Technical University of Munich, Munich, Germany; 4 Institute for Synthetic Biomedicine, Helmholtz Munich, Neuherberg, Germany; 5 Department of Bioscience, TUM School of Natural Sciences, Technical University of Munich, Garching, Germany; 6 Institute of Metabolism and Cell Death, Helmholtz Munich, Neuherberg, Germany; 7 TUM School of Medicine, Technical University of Munich, Munich, Germany

**Keywords:** Bioluminescence imaging, Microscopy, Cellular imaging

## Abstract

Bioluminescence is an attractive alternative to fluorescence for live-cell imaging; however, its low intensity has prevented widespread adoption. Specialized microscopes compensate by sacrificing spatial resolution, field of view and dynamic range—constraints imposed by the highest-sensitivity camera to date: the electron-multiplying charge-coupled device. Recently, quanta image sensor (QIS) technology has emerged for low-light imaging. Here, we show that a commercial QIS camera has exceptional sensitivity; however, its sensor dimensions necessitate a microscope designed to maximize its properties. We introduce a Keplerian-telescope-inspired microscope setup that, with the QIS, results in modestly improved signal-to-noise ratios at substantially higher spatial resolution, field of view and dynamic range, relative to the state of the art. The telescopic design also confers modularity, enabling multimodal imaging with epifluorescence. The ‘QIScope’ makes bioluminescence a viable tool for technically challenging live-cell experiments such as monitoring intracellular and extracellular vesicles simultaneously and the dynamics of low-abundance proteins.

## Abstract (web summary)

Bioluminescence imaging with enhanced sensitivity, resolution and dynamic range is enabled by a camera based on quanta image sensor technology in combination with a dedicated unconventional microscope design. The capabilities of the QIScope are demonstrated in live imaging of extracellular vesicles or low-abundance proteins.

## Main

Live-cell imaging is a pillar of biological research because of the utility of spatially and temporally monitoring internal and external cellular processes. To date, fluorescence is the imaging modality of choice due to the advantages of fluorescent dyes and proteins including specific targeting, genetic encoding, multicolor multiplexing and analyte sensing^1–3^. However, the use of an excitation light source can lead to high background, low dynamic range, phototoxicity and probe photobleaching, limiting the scope of measurement sensitivity, sample choice and experiment duration^4–7^. An attractive alternative to fluorescence is bioluminescence, which uses chemical, rather than photonic, energy to produce light^8^. Hence, bioluminescent reporters circumvent the problems of fluorescence, while still offering analogous functionalities as their fluorescent counterparts^9–13^.

The main limitation of bioluminescence remains its low emission intensity. Despite advancements in proteins and substrates^8,14–17^, enzymatic turnover inevitably restricts bioluminescence photon emission to rates orders of magnitude lower than possible by photon absorption and emission in fluorophores^10,18,19^. Therefore, bioluminescence imaging of cells generally requires specialized microscopes optimized for photon collection, transmission, image formation and detection, at the expense of spatial and temporal resolution, field of view (FOV) and dynamic range^20,21^. Unfortunately, in contrast to probes, advancements in bioluminescence microscopes have been relatively stagnant largely due to the constraints of the leading detector technology for low-light imaging: the electron-multiplying charge-coupled device (EMCCD)^22^. As a result, the advantages of bioluminescence often do not outweigh its disadvantages when researchers aim to resolve structures and dynamics at the subcellular level.

Here, we report the use of a recently developed detector technology in bioluminescence microscopy: the QIS^23,24^. Moreover, we have reconsidered the design of bioluminescence microscopes and introduced an optical setup, inspired by the Keplerian telescope, which maximizes the unique properties of the QIS camera. In direct comparison with the commercial state-of-the-art Olympus LV200 bioluminescence microscope equipped with an EMCCD, our ‘QIScope’ captures images of cellular bioluminescence with modestly improved signal-to-noise ratio (SNR) at substantially higher spatial resolution, FOV and dynamic range. These features enable challenging experiments such as the simultaneous imaging of intracellular and extracellular vesicles (EVs) or low-abundance proteins labeled with bioluminescent reporters, while maintaining straightforward integration with conventional epifluorescence imaging.

## Results

### Benchmarking the QIS against leading sCMOS and EMCCD cameras

A bioluminescence microscope fundamentally consists of three components: an objective lens for light collection, a ‘tube’ lens for image formation, and a camera for light detection. These individual components are widely considered to be fully optimized for low-light imaging. For example, photon collection efficiencies of high numerical aperture (NA) immersion objective lenses have nearly reached their theoretical maximum. Likewise, modern optical lenses permit near-unity transmission efficiencies. Despite efforts to optimally select and reconfigure these optics^25,26^, the limiting factor in bioluminescence microscopy remains the performance of the detector.

For two decades, EMCCD cameras have been the leading detector for imaging weakly emissive samples ranging from single atoms and molecules, to cellular reporters, to stars^27–32^. This capability arises from their ability to apply large enough gain that even single photons can be resolved when the amplified signal exceeds the amplified noise^33,34^. More recently, the scientific complementary metal–oxide–semiconductor (sCMOS) camera has emerged as a potential alternative, achieving sensitivity primarily through noise suppression rather than signal amplification, while also offering higher resolution due to smaller pixels, larger FOV due to larger sensor sizes and larger dynamic range due to lower gain^35,36^. Despite these advantages, the sCMOS camera has still generally underperformed the EMCCD camera in photon-starved conditions^22,37^.

The QIS represents the next step in the evolution of high-sensitivity sCMOS detectors. By shrinking the pixel size and increasing conversion gain, exceptionally low noise is achieved, permitting single-photon detection on a CMOS chip^23,24,38^. Here, we apply a commercial QIS camera to bioluminescence microscopy: the QIS16TS developed by Gigajot Technology.

We begin by benchmarking the QIS16TS (‘QIS’) against two leading cameras used in high-sensitivity microscopy: the Andor iXon Ultra 897 (‘EMCCD’) and the Hamamatsu ORCA-Fusion BT (‘sCMOS’; Supplementary Table 1). Measurements were performed on the same home-built epifluorescence microscope setup under identical low-intensity excitation (1 nW) and integration times (2 s). However, due to pixel size variations between cameras, neutral-density (ND) filters were placed in the emission path to approximate the same photon flux per pixel for each camera (Supplementary Fig. 1a).

**Fig. 1:**
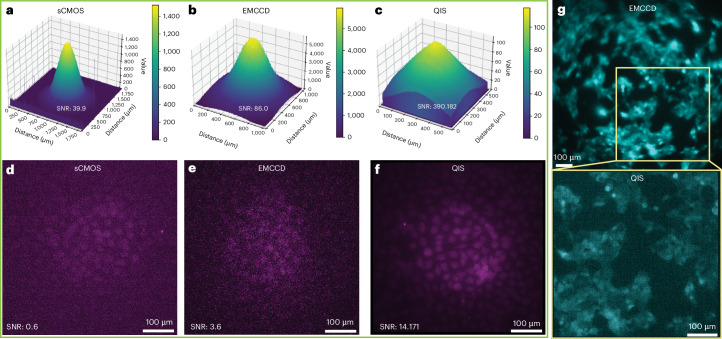
Comparison of sCMOS, EMCCD and QIS cameras. **a**–**c**, Three-dimensional plots of the same fluorescent slide measured under identical conditions (Supplementary Fig. 1a), but with the sCMOS (**a**), EMCCD (**b**) and QIS (**c**) cameras. ND filters were used to normalize the photon flux per pixel. Exposure time, 2 s. **d**–**f**, Fluorescence images of MEFs labeled with quantum dots acquired by the sCMOS (**d**), EMCCD (**e**) and QIS (**f**) cameras, under identical conditions and adjusted for photon flux per pixel. Exposure time, 10 s. Effective pixel size of **a** and **d**, 812.5 nm. Effective pixel size of **b** and **e**, 2,000 nm. Effective pixel size of **c** and **f**, 137.5 nm. ND filters used: sCMOS, ND1.5 (NE10B-A and NE05B-A, Thorlabs); EMCCD, ND 2.4 (NE20B-A and NE04B-A, Thorlabs). No ND filters were used for the QIS. **g**, Sensor size comparison of the EMCCD and QIS when using the same optical setup to image the same sample of bioluminescent cells (same sample as in Extended Data Fig. 4). Exposure time, 100 s. Representative results are shown from two to three independent experiments.

Our first ‘sample’ was a fluorescent slide. As expected, the SNR obtained by the EMCCD (electron multiplication (EM) gain = 300) was more than double that of the sCMOS (Fig. 1a,b). However, we found that the SNR of the QIS camera was ~4.5-fold that of the EMCCD and nearly 10-fold that of the sCMOS under similar photons-per-pixel conditions (Fig. 1c). These results were recapitulated in measurements of mouse embryonic fibroblasts (MEFs) labeled with colloidal quantum dots still under the same average photons-per-pixel conditions and zoomed into the same FOV (Fig. 1d–f).

**Extended Data Fig. 1:**
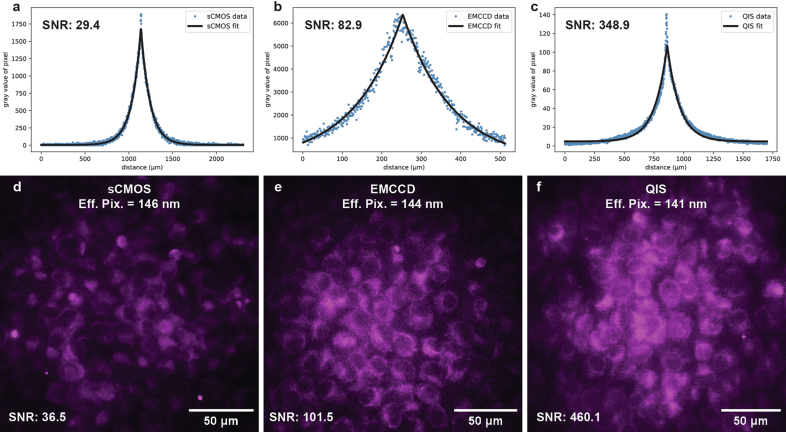
Comparison of sCMOS, EMCCD, and QIS Cameras at Same Effective Pixel Size. **a–c**, Intensity profiles of the same fluorescent slide measured under identical excitation conditions and the same effective pixel size (Supplementary Table 2), but with the sCMOS (**a**), EMCCD (**b**) and QIS cameras (**c**), respectively. Exposure time: 1 s. Laser wavelength: 470 nm. Laser intensity: 1.05 nW (Average: $\sim 3\times {10}^{-5}W/{{cm}}^{2}$, Peak: $\sim 1\times {10}^{-2}W/{{cm}}^{2}$). The power was measured at the sample plane and the laser spot size was determined from a fit to the beam profile at the sample using the 1/e² intensity values. Longpass filter: 500 nm. Beamsplitter: BSN10R, Thorlabs. Sample: Thorlabs FSK4. **d–f**, Fluorescence images of mouse embryonic fibroblasts labeled with quantum dots acquired by the sCMOS (**d**), EMCCD (**e**), and QIS cameras (**f**) respectively, under identical excitation conditions. Exposure time: 500 ms. Laser wavelength: 460 nm. Laser intensity: 0.67 μW (Average: $\sim 4\times {10}^{-3}W/{{cm}}^{2}$, Peak: $\sim 2W/{{cm}}^{2}$). The power was measured and processed in the same way as **a**—**c**. Longpass filter: FELH0500, Thorlabs. Beamsplitter: BSN10R, Thorlabs. Additional setup details are found in Supplementary Table 2. Representative results are shown from one to two independent experiments.

The photon flux per pixel can also be normalized by adjusting the image magnification using different lenses, rather than via ND filters. Three distinct optical setups were built, each featuring magnifications tuned to achieve the same ‘effective pixel size’ for each camera (Supplementary Table 2). For both the fluorescent slide and quantum-dot-labeled MEFs, the relative SNR between the three cameras mirrored the results of Fig. 1 (Extended Data Fig. 1 and Supplementary Table 3).

These results highlight how, under similar photon-per-pixel levels, the QIS can capture images of higher quality than both the sCMOS and EMCCD. In practice, however, the small pixel size of the QIS introduces two major liabilities: low photon flux per pixel and small chip size. That is, if the QIS were to directly replace the sCMOS or the EMCCD on an identical microscope setup, it would receive nearly 35-fold fewer photons per pixel than the sCMOS and over 210-fold fewer photons per pixel than the EMCCD, resulting in a much lower overall SNR. Moreover, the limited sensor size of the QIS (20.25 mm^2^) results in a maximum FOV more than 10-fold smaller than the sCMOS (224.28 mm^2^) and more than 3-fold smaller than the EMCCD (67.11 mm^2^; Fig. 1g). To take full advantage of the QIS for microscopy, the optical setup design must be reconsidered.

### Design and benchmarking of telescopic microscope with QIS

Despite the excellent detection capabilities of the QIS, its small sensor dimensions demand a microscope that can both increase the photon flux per pixel, while still capturing a large FOV. Intuitively, this means shrinking/demagnifying the image to fit onto a small sensor chip, which consequently also squeezes more light onto each pixel. This inverse relationship between photons per area, or image brightness (*B*), and effective magnification (*M*_eff_) can be expressed according to equation (1):

1 $$B\propto \frac{{\rm{NA}}^{2}}{{M}_{\rm{eff}}^{2}}$$

Equation (1) suggests two approaches for increasing the brightness of images: increasing photon collection (higher NA) or decreasing effective magnification (lower *M*_eff_). As previously mentioned, the NA of modern immersion objectives cannot be improved much. However, *M*_eff_ can be reduced by the combined magnifications of the objective lens and tube lens. This is the approach taken by the commercial Olympus LV200 bioluminescence microscope, which uses a specialized tube lens to lower *M*_eff_ (5-fold reduction) and boost the photon flux per pixel^20^. However, given the much smaller pixel size of the QIS sensor relative to other scientific cameras, an unprecedented reduction in *M*_eff_ is needed to achieve a sufficiently high photon flux per pixel (SNR).

**Extended Data Fig. 2:**
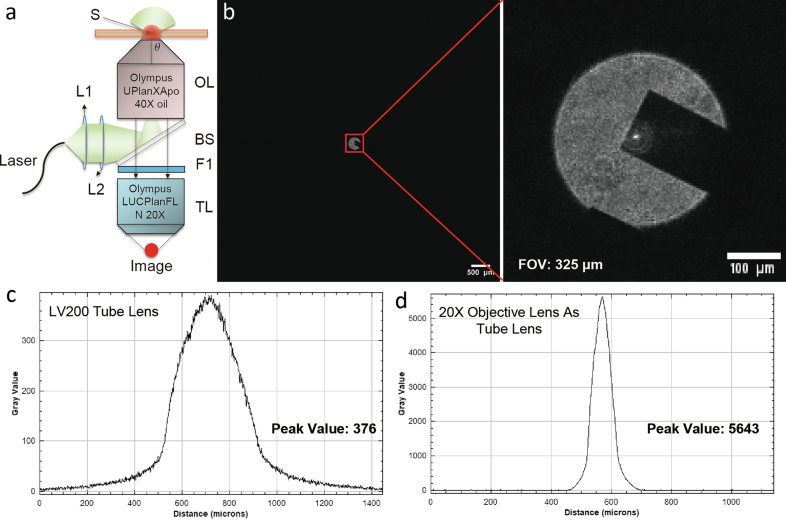
Properties of the 40×/20× Two-objective Microscope. **a**, Schematic of the 40×/20× two-objective microscope. L1: Lens 1, L2: Lens 2, S: Sample, OL: Objective lens, BS: Beamsplitter (BSN10R, Thorlabs), F1: 500 nm Longpass Filter, TL: Tube lens. Distance between OL and TL: ~30 mm. Representative results are shown from two independent experiments. **b**, Transmission illumination image of a resolution target shows strongly restricted field of view (FOV = 325 μm). **c, d**, Intensity profiles measured from a fluorescent slide under the identical setup except with the tube lens exchanged: LV200 tube lens (**c**) and 20× objective lens as tube lens (**d**).

Our efforts to adopt a commercially available optic as the tube lens for the QIS proved unsuccessful. For example, the combination of a 40× oil-immersion objective lens and a 20× objective as the tube lens (*M*_eff_ = 2×, 20-fold reduction) indeed resulted in a much higher SNR (Extended Data Fig. 2). However, this SNR increase came at the expense of a strongly restricted FOV, rendering this microscope setup largely impractical for cell studies. Our inability to find a combination of two objective lenses that gave sufficiently low *M*_eff_, without also severely compromising FOV, called into question the viability of the conventional two-lens microscope setup for use with the QIS camera.

In response to this challenge, we engineered an optical microscope setup that simultaneously increases photons per pixel (smaller *M*_eff_) without sacrificing FOV. In conventional bioluminescence microscopes, the objective and tube lenses are positioned as close as possible to maximize light transmission, minimize stray background light and maximize FOV^25,26^. However, as evidenced by the 40×/20× two-objective microscope (Extended Data Fig. 2), the much smaller back aperture of the tube lens objective constricts the image FOV, even in the absence of lens separation.

**Fig. 2:**
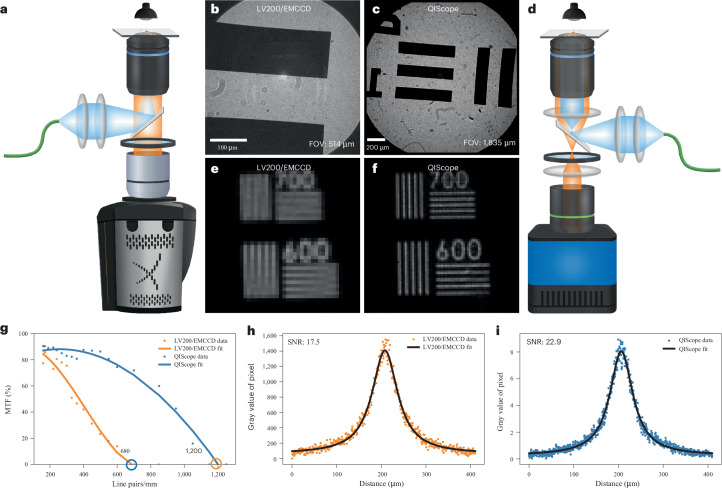
Benchmarking the QIScope against the LV200/EMCCD. **a**,**d**, Schematic of LV200/EMCCD with a 100× oil-immersion objective lens (**a**) and QIScope with a 40× oil-immersion objective lens, a 20× objective lens as the tube lens and a Keplerian telescope in between (**d**). For epifluorescence experiments, an emission filter was placed within the telescope. **b**,**c**, Comparison of the LV200/EMCCD (**b**) and QIScope (**c**) FOVs gives 514 μm and 1,835 μm, respectively. **e**,**f**, The resolution test target imaged at 600 and 700 parallel lines per mm (pl mm^−1^) by the LV200/EMCCD (**e**) and QIScope (**f**), respectively. **g**, Comparison of the MTFs for the two microscopes. Polynomial fits to data points are guides for the eye. The resolution limit of the LV200/EMCCD is 680 pl mm^−1^ and of the QIScope is 1,200 pl mm^−1^. **h**,**i**, Intensity profiles with Lorentzian fits measured from a fluorescent slide under identical excitation conditions for LV200/EMCCD (**h**; SNR = 17.5) and QIScope (**i**; SNR = 22.9). Exposure time, 10 s. Effective pixel size of LV200/EMCCD, 800 nm. Effective pixel size of QIScope, 423 nm.

Counterintuitively, this ‘vignetting’ effect can be avoided by separating the objective lens and tube lens and inserting a Keplerian telescope in between (Fig. 2d). This ‘telescope-within-a-microscope’ effectively reshapes the output of the objective lens to match the width of the tube lens back aperture, resulting in substantial image size reduction (*M*_eff_ = 2.6×, 15.4-fold reduction), while still capturing the complete FOV. Importantly, this configuration maintains a high-quality objective lens as the final optical component (‘tube lens’) before the detector, preserving image fidelity^39^. However, due to the small working distance of the high-NA tube lens objective, the QIS camera could only be used upon removal of the camera flange and careful stage micrometer positioning. We name the combined telescopic microscope setup with the QIS camera, the ‘QIScope’ (Fig. 2d).

**Extended Data Fig. 3:**
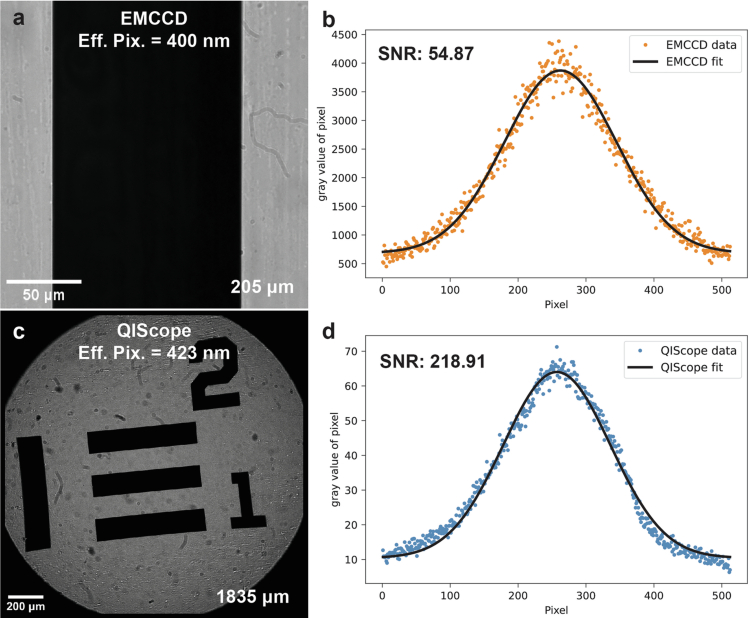
Benchmarking the QIScope against the EMCCD at the Same Effective Pixel Size. **a, c**, Comparison of the EMCCD (**a**) and QIScope (**c**) fields of view (FOV) gives 205 μm and 1835 μm, respectively. **b, d**, Intensity profiles with Gaussian fits measured from a fluorescent slide under identical excitation conditions for the EMCCD (b, SNR = 54.87) and QIScope (d, SNR = 218.91), respectively. Exposure time: 0.5 s. Laser wavelength: 470 nm. Laser intensity: 0.8 μW (Average: $\sim 5\times {10}^{-3}W/{{cm}}^{2}$. Peak: $\sim 2W/{{cm}}^{2}$). The power was measured at the sample plane and the laser spot size was determined from a fit to the beam profile at the sample using the 1/e^2^ intensity values. Longpass filter: FELH0500, Thorlabs. Beamsplitter: BSN10R, Thorlabs. Sample: Thorlabs FSK4. Additional setup details are found in Supplementary Table 4.

We first compared our QIScope to a home-built microscope equipped with the EMCCD operating at the same effective pixel size (Supplementary Table 4). We found that the QIScope possessed a ~9-fold greater FOV under transmission brightfield imaging, with ~4-fold greater SNR for a fluorescent slide under the same excitation intensity in an epifluorescence configuration (Extended Data Fig. 3). This shows that the QIScope configuration maintains the advantages of the QIS while achieving a high FOV.

Importantly, we benchmarked our QIScope against a home-built version of the Olympus LV200 bioluminescence microscope equipped with a 100× oil-immersion objective, the LV200 tube lens and the iXon 897 EMCCD camera (‘LV200/EMCCD’; Fig. 2a). Here, the 100× objective was used to maximize both the photon collection (NA = 1.45) and spatial resolution of the LV200 system. First, using transmission illumination, we found that the QIScope possessed a ~3.6-fold larger FOV than the LV200 (Fig. 2b,c). This corresponds to a nearly 13-fold increase in viewable area.

Next, using a resolution test target, we found that the QIScope could clearly resolve narrowly spaced lines that the LV200/EMCCD could not (Fig. 2e,f). By imaging a series of line spacings, we calculated the modulation transfer function (MTF) and found that the image resolution of the QIScope was 1.77 times that of the LV200/EMCCD (Fig. 2g and Supplementary Fig. 2). This is in good agreement with the theoretical ratio of 1.89.

Finally, we compared the sensitivity of the two systems by measuring a fluorescent slide under the same excitation intensity in an epifluorescence configuration (Fig. 2h,i). Here, the SNR of the QIScope was ~31% greater than that of the LV200/EMCCD despite the QIS receiving far fewer photons per pixel than the EMCCD. In summary, a direct comparison of the QIScope with the LV200/EMCCD microscope shows that the QIScope can measure images with moderately improved SNR at substantially higher spatial image resolution and FOV.

### Imaging low-intensity bioluminescence from live cells

To determine if the improved performance of the QIScope relative to the LV200/EMCCD translates to bioluminescence imaging of live cells, we measured the weak bioluminescence of the exon-specific isoform expression reporter system (‘EXSISERS’) in exon 10 of the alternatively spliced gene microtubule-associated protein tau (*MAPT*). The disease-associated exon 10 is rarely included in wild-type *MAPT*, leading to low expression of the NanoLuc-luciferase (NLuc) reporter^40^.

**Extended Data Fig. 4:**
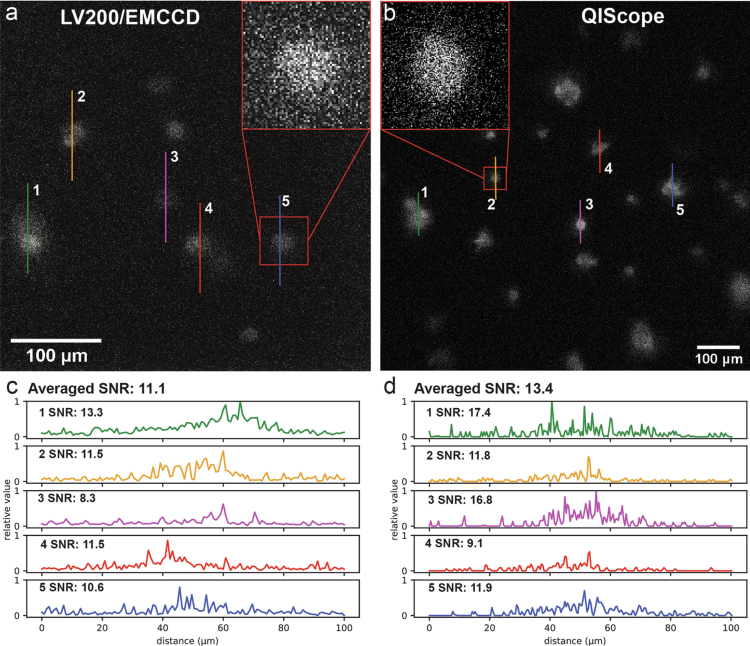
Imaging Weak Live-Cell Bioluminescence. **a, b**, Images of EXSISERS cells obtained by the LV200/EMCCD (a) and QIScope (b). The area of the red boxed inset is 50 × 50 μm. Exposure time: 10 s. Substrate: Nano-Glo Vivazine Substrate. **c, d**, Intensity line profiles over cells/clusters marked in **a**,**b** are plotted for the LV200/EMCCD and QIScope, respectively. Peak SNR values are given for each trace, along with an average SNR over all five traces. Effective pixel size of LV200/EMCCD: 800 nm. Effective pixel size of Qiscope: 423 nm. Representative results are shown from three independent experiments.

**Extended Data Fig. 5:**
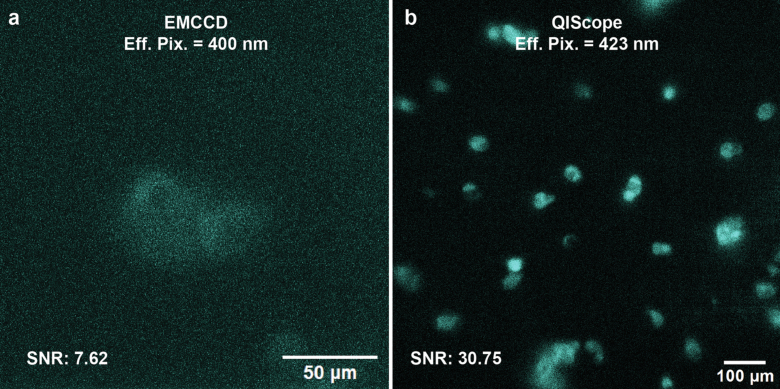
Imaging Weak Live-Cell Bioluminescence at the Same Effective Pixel Size. **a, b**, Image(s) of EXSISERS cells obtained by the EMCCD (a, SNR = 7.62) and QIScope (b, SNR = 30.75) at the same effective pixel size. SNR values obtained from intensity line profiles of cell clusters. Exposure time: 10 s. Substrate: Nano-Glo Vivazine Substrate. Representative results are shown from two independent experiments.

A direct comparison of EXSISERS cells imaged by the LV200/EMCCD microscope and our QIScope show the much larger FOV and higher spatial resolution of the QIScope (Extended Data Fig. 4 and Supplementary Fig. 1c). Intensity cross-sections of five cells/clusters show that the QIScope measured a higher ‘peak’ SNR value (31% higher) as well as a higher ‘average’ SNR value (21% higher) relative to the LV200/EMCCD system. Measurements of these cells using the QIScope and a microscope equipped with the EMCCD at the same effective pixel size again reflect the relative SNR advantage of the QIS (Extended Data Fig. 5). We note, however, that the QIS appears to underperform at very long integration times, possibly from accumulation of dark current noise (Supplementary Fig. 3). Overall, these results indicate that the FOV, spatial image resolution and SNR benchmarking results of Fig. 2, obtained with transmission and fluorescence imaging, indeed translate over to live-cell bioluminescence imaging.

### Benchmarking bioluminescence from EVs

The exceptional FOV, spatial image resolution and sensitivity of the QIScope is maintained for bioluminescence imaging. However, for many research questions, an imaging system must meet additional demands, such as high spatiotemporal resolution and dynamic range, to resolve subcellular processes. Therefore, we challenge the QIScope’s capabilities and showcase its utility by measuring cells exhibiting bioluminescence spanning orders of magnitude in space, time and intensity.

EVs, membrane-coated biological particles originating from cells for cargo transport and intercellular communication, encompass a diverse array of structures classified by the cellular process that generated them^41,42^. For example, exosomes originate from inward budding of late endosomes to form intraluminal vesicles within multivesicular bodies (MVBs), which later fuse with the plasma membrane to release exosomes extracellularly^43^. This is in contrast to ectosomes/microvesicles, which are directly formed through outward budding of the plasma membrane. Migrasomes are another class of EVs that constitute trails left behind from retraction fibers of migrating cells^44^.

Even within a class, EVs exhibit considerable heterogeneity in size (ranging from tens of nanometers to microns), localization (intracellular to extracellular) and dynamics (seconds to hours). These properties make the global imaging of EVs from live cells challenging, even for established modalities such as fluorescence^45^. It is perhaps not surprising that few studies have used bioluminescence for cellular EV studies. As we show below, the current state-of-the-art LV200/EMCCD microscope is insufficient for EV imaging by bioluminescence, but the QIScope opens new possibilities for these studies.

**Fig. 3:**
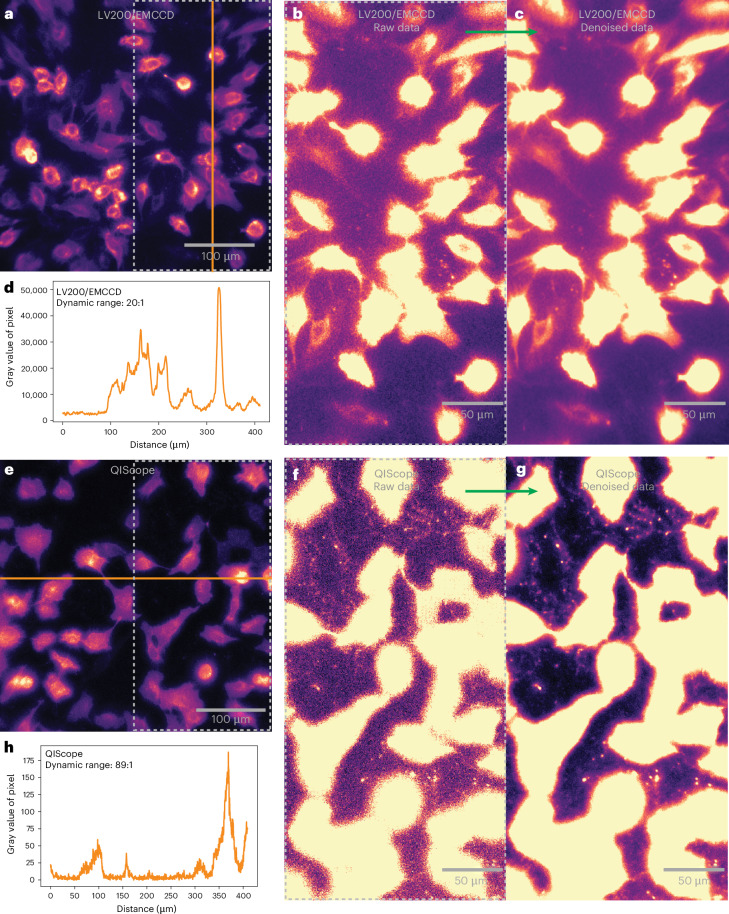
Imaging of EV bioluminescence by the LV200/EMCCD and QIScope. **a**,**e**, Bioluminescence images at low contrast of MEFs expressing NLuc–CD63 acquired under identical conditions by the LV200/EMCCD (**a**) and QIScope (**e**). Exposure time, 2 s. Substrate, Nano-Glo Live Cell Substrate. **b**,**f**, Images of the gray dashed boxed area in **a** and **e** under high-contrast settings. **c**,**g** Denoised versions of **b** and **f**. **d**,**h**, The intensity profiles of the orange lines in **a** and **e** are plotted along with the dynamic range. Effective pixel size of LV200/EMCCD, 800 nm. Effective pixel size of QIScope, 423 nm. Representative results are shown from three independent experiments.

In Fig. 3, we present a direct comparison of the LV200/EMCCD imaging system compared to our QIScope for imaging MEFs expressing NLuc N-terminally fused to CD63, a tetraspanin protein widely used as an exosomal marker^46^. Under low-contrast settings, images taken on both microscopes appeared similar, with robust intracellular intensity (Fig. 3a,e). Under high-contrast settings, however, the same images were qualitatively different in the extracellular space (Fig. 3b,f). Both microscopes were capable of detecting large EVs between cells; however, only the QIScope could resolve the network of linear trails and pockets of smaller EVs previously observed by fluorescence and electron microscopy^47^. Moreover, only the QIScope was capable of resolving the diffusion of smaller EVs around the cells (Supplementary Videos 1 and 2). We note that considerable effort was needed to find any sign of EVs between cells using the LV200/EMCCD, whereas EVs were found with ease using the QIScope. The QIScope’s larger FOV also helped to image across more cells and identify more EVs.

**Extended Data Fig. 6:**
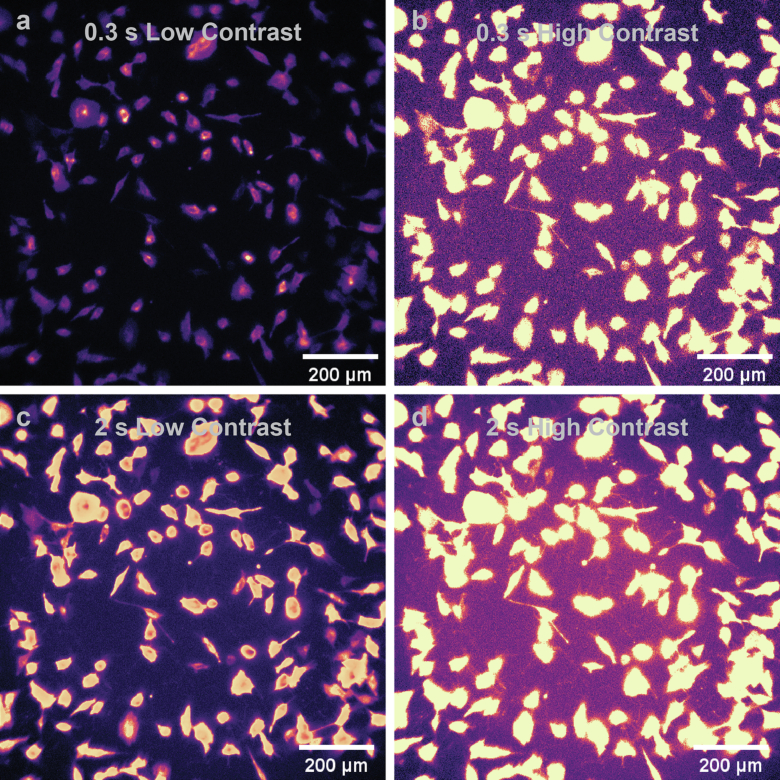
Imaging Intracellular and Extracellular Vesicles on LV200/EMCCD with 40× Objective Lens. **a, b** Imaging intracellular (**a**) and extracellular (**b**) vesicles from NLuc-CD63 cells with different contrast settings for the same image under 0.3 s exposure time. **c, d** Imaging intracellular (**c**) and extracellular (**d**) vesicles with different contrast settings for the same image under 2 s exposure time. EV trails between cells are visible with the 40× objective and longer integration time. Substrate: Nano-Glo Live Cell Substrate. Effective pixel size: 2000 nm. Representative results are shown from five independent experiments.

The difference in EV imaging between the LV200/EMCCD and the QIScope in Fig. 3b,f can largely be attributed to the performance gap between the two systems. The higher sensitivity and spatial image resolution of the QIScope allows it to resolve trails and particles that would otherwise appear as faint smears. This performance gap is magnified by the use of image denoising algorithms such as Noise2Info (for single images)^48^ and Noise2Noise (for movies)^49^. When applied to the images from the QIScope, denoising substantially improves the visibility of trails and particles in the extracellular space (Fig. 3f,g). However, despite our best efforts, we were unable to resolve additional EVs in images taken with the LV200/EMCCD even with denoising (Fig. 3b,c), indicating that EV information content is lacking. With the lower-magnification 40× objective, resulting in higher SNR, the LV200/EMCCD could eventually resolve extracellular EV trails (Extended Data Fig. 6) but at the cost of even lower spatial resolution and limited intracellular information due to pixel saturation.

From these data, two additional features of the QIScope stand out. First, the temporal resolution of the QIScope is sufficiently high (2 s) to resolve subcellular dynamics, such as EV diffusion (Supplementary Video 2). Second, the dynamic range of the QIScope exceeds that of the LV200/EMCCD by a factor of nearly 4.5 due to EMCCDs being prone to saturation (Fig. 3d,h and Supplementary Fig. 4). This permits the simultaneous measurement of both sparse extracellular, and dense intracellular, structures that cannot be achieved on the LV200/EMCCD system. The demand for high dynamic range has limited even fluorescence measurements, prompting the use of pH-sensitive reporters to distinguish between intracellular and extracellular vesicles^47,50^. However, with bioluminescence imaging on the QIScope, images of comparable quality to fluorescence can be obtained without the use of specialized fluorescent proteins, while still maintaining the aforementioned advantages of bioluminescence.

### Analyzing EVs via bioluminescence

The QIScope enables live-cell EV studies that were previously not possible via bioluminescence. We illustrate below several use cases by analyzing static and dynamic measurements of MEFs expressing NLuc targeted to different subcellular locations: the cytoplasm (untagged NLuc, or ‘Cyto–NLuc’), the plasma membrane (myristoylation/palmitoylation sequence of the tyrosine kinase Lck N-terminally fused to NLuc, or ‘Myrpalm–NLuc’) and exosomes/MVBs (NLuc N-terminally fused to CD63, or ‘NLuc–CD63’; Supplementary Fig. 5)^51–53^.

**Extended Data Fig. 7:**
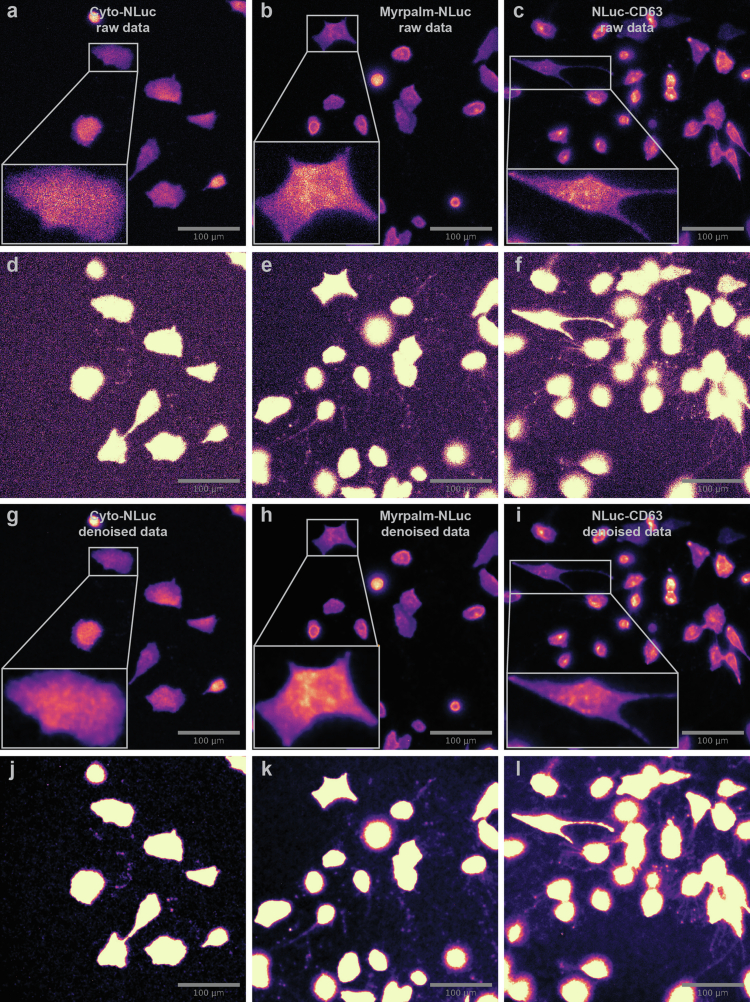
Imaging Different Types of Intracellular and Extracellular Vesicles using the QIScope. **a–c**, Raw bioluminescence images of Cyto-NLuc, Myrpalm-NLuc, and NLuc-CD63 MEFs under low contrast settings. Exposure time: 10 s. Substrate: Nano-Glo Vivazine substrate. **d–f**, The same images as **a**–**c**, respectively, under high-contrast settings. **g–i**, Denoised versions of **a**–**c**, respectively. **j–l**, Denoised versions of **d**–**f**, respectively. Effective pixel size: 423 nm. Representative results are shown from four independent experiments.

Low-contrast, ‘static’ images of the three cell lines reflect the differences in intracellular NLuc targeting (Extended Data Fig. 7a–c,g–i). Here, the Cyto–NLuc signal was homogeneously distributed throughout the cytoplasm, the Myrpalm–NLuc signal was distributed less uniformly due to localization to various membranous compartments, and the NLuc–CD63 signal was localized to puncta throughout the cell, likely lysosomes and MVBs, as previously observed^51^.

The same images under high-contrast image settings reveal NLuc emission in the extracellular space (Extended Data Fig. 7d–f,j–l). As expected, Cyto–NLuc cells showed few, but relatively large spots of signal, reminiscent of cellular debris and microvesicles passively incorporating cytosolic proteins. In contrast, Myrpalm–NLuc and NLuc–CD63 cells exhibited pronounced extracellular signal with Myrpalm–NLuc signal generally localized to larger puncta and NLuc–CD63 signal localized to smaller puncta along linear trails. These differences may reflect the tendency of these markers to localize to different subdomains of extracellular membranes and vesicle structures^54^.

Dynamic imaging further discriminated Myrpalm–NLuc from NLuc–CD63 at both the intracellular and extracellular levels. Intracellularly, we could follow the general movement of the plasma membrane in a Myrpalm–NLuc cell undergoing macropinocytosis/endocytosis (Supplementary Video 3 and Extended Data Fig. 7b,h), while a NLuc–CD63 cell showed numerous smaller puncta, likely MVBs, undergoing directed movement (Supplementary Video 4 and Extended Data Fig. 7c,i).

**Extended Data Fig. 8:**
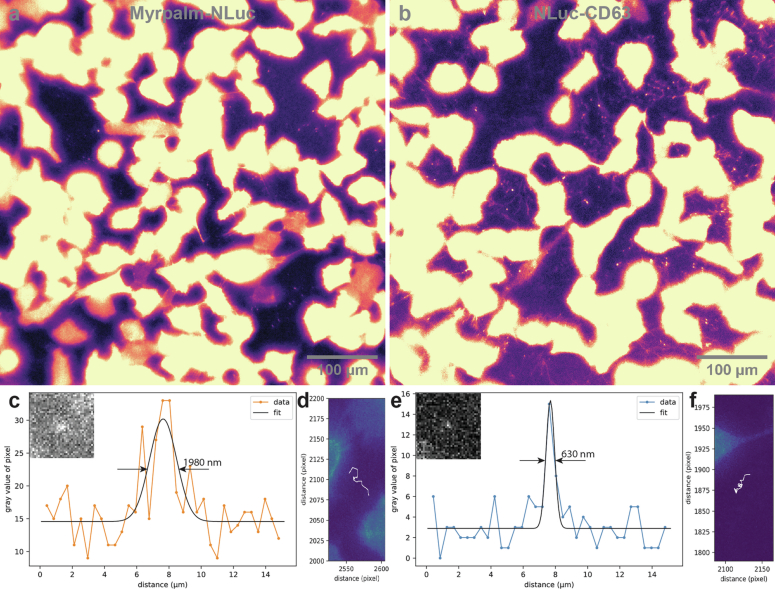
Comparison of Two Types of Extracellular Vesicles. **a, b**, Images of Myrpalm-NLuc and NLuc-CD63 cells with the Nano-Glo Live Cell Substrate on the QIScope. Exposure time: 2 s. Substrate: Nano-Glo Live Cell Substrate. **c**,**e**, Snapshots and line profiles with Gaussian fits of single diffusing particles derived from Myrpalm-NLuc and NLuc-CD63 samples, respectively. Particle sizes are indicated as the full-width at half-maximum of the Gaussian fit. **d**,**f**, Diffusion traces (white lines) of the EVs in c and e, respectively, overlaid onto their images. See also Supplementary Videos 5 and 6. Effective pixel size: 423 nm. Representative results are shown from three independent experiments.

Extracellularly, single EVs could be resolved using the brighter Nano-Glo Live Cell Substrate, enabling single-particle tracking (Extended Data Fig. 8a,b). We highlight here the tracking of two individual particles found near Myrpalm–NLuc (Supplementary Video 5) and NLuc–CD63 cells (Supplementary Video 6). Despite the Myrpalm EV being >2.5 times larger in diameter than the CD63 EV (1,980 nm versus ≤630 nm, due to the resolution limit; Fig. 2g), their average two-dimensionally projected diffusion velocities were similar (1.3 µm s^−1^ versus 1.4 µm s^−1^). Given that classical Brownian diffusion predicts faster velocities for smaller EVs, the observed similar speeds for these EVs suggest restricted diffusion of EVs within the extracellular matrix, with differences possibly attributed to matrix–EV interactions^55^.

Finally, we highlight the dynamic range of our system and bioluminescence, which enables hybrid measurements monitoring both the intracellular and extracellular space. One example, observed in a Myrpalm–NLuc cell, shows the movement of the cell’s membrane extension to find and attach to a surface-bound EV (Supplementary Video 7), consistent with reports of migrasome internalization via filopodia^47,56^. Another example is the transfer of CD63-labeled cargo between two cells through a cellular tunneling nanotube (Supplementary Video 8), which, to our knowledge, has previously only been observed by fluorescence microscopy^57,58^. In summary, the QIScope enables the use of bioluminescence for technically challenging measurements of small, faint and dynamic subcellular structures both inside and outside cells at the same time.

### Multimodal bioluminescence and epifluorescence imaging

The QIScope enables a range of live-cell bioluminescence studies that were previously not possible on the LV200/EMCCD system. We show here an additional feature of the QIScope missing from the conventional system: integration of epifluorescence. The LV200 system integrates fluorescence with bioluminescence imaging using transmission excitation, rather than epifluorescence^59^. This approach maximizes the FOV by keeping the microscope objective lens as close to the tube lens as possible, but at the cost of much higher background from excitation light bleed-through^25,26^. However, in the case of the QIScope, the telescope between the objective lens and tube lens creates ample room for a beamsplitter without sacrificing FOV (Fig. 2d). The integration of epifluorescence enables high-SNR multimodal measurements of, for example, two specific targets using orthogonal labels.

**Fig. 4:**
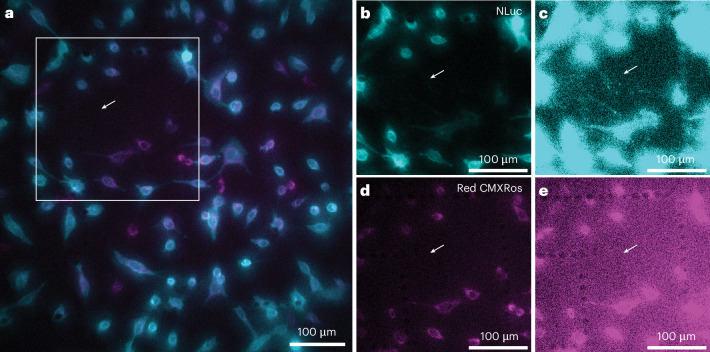
Multimodal imaging on the QIScope: bioluminescence and epifluorescence. **a**, The bioluminescence of NLuc–CD63 (turquoise) and the fluorescence of MitoTracker Red CMXRos dye (purple) of the same MEFs imaged sequentially on the QIScope. **b**,**c**, Isolated bioluminescence signal from the white square in **a** at low (**b**) and high (**c**) image contrast. EVs were observed at high contrast in bioluminescence (arrow). Exposure time, 2 s. Substrate, Nano-Glo Live Cell Substrate. **d**,**e**, Isolated fluorescence signal from the same white square in **a** at low (**d**) and high (**e**) image contrast. No EVs were observed by fluorescence (arrow). Regularly spaced dots in **c** and **e** correspond to imprinted grids on the chamber surface. Exposure time, 0.5 s. Effective pixel size, 423 nm. Representative results are shown from five independent experiments.

In Fig. 4, we give an example of multimodal imaging by bioluminescence and fluorescence using the epifluorescence-modified QIScope. Here, the same MEFs were imaged with NLuc–CD63 bioluminescence and mitochondria labeled with the MitoTracker Red CMXRos fluorescent dye (Fig. 4). At low imaging contrast, it was evident that the intracellular localization of CD63 and mitochondria were different. At high contrast, EVs labeled with NLuc–CD63 were visible between cells (Fig. 4c), as in Fig. 3 and Extended Data Figs. 6–8. The same particles showed no MitoTracker fluorescence, indicating no mitochondria in the EVs, as expected (Fig. 4e). These measurements, taken seconds apart due to manual insertion of the fluorescence emission filter, highlight how nearly simultaneous live-cell monitoring of mitochondria and EVs could be performed, for example, as a function of the cellular metabolic state or response to drugs.

### Advantageous use cases for bioluminescence over fluorescence

The dual bioluminescence and epifluorescence capability of the QIScope offers an opportunity to explore scenarios where bioluminescence may hold distinct advantages over fluorescence in live-cell imaging. Below, we compare the two modalities using several canonical benchmarks, culminating in a long-duration bioluminescence measurement of a low-abundance protein in live cells.

**Fig. 5:**
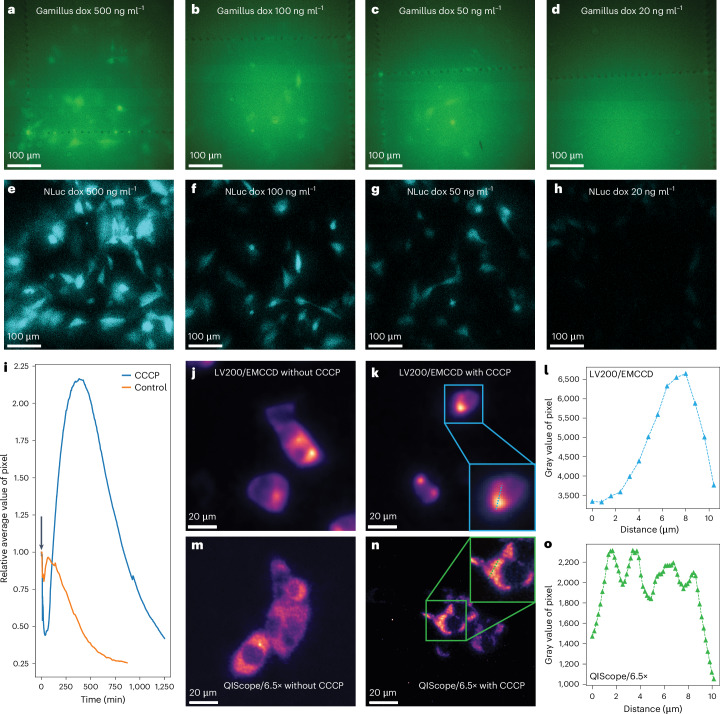
Comparing bioluminescence and fluorescence sensitivity and observing PINK1 subcellular dynamics. **a**–**d**, The fluorescence signal from MEFs expressing a Gamillus–NLuc fusion protein imaged at different dox levels. Exposure time, 0.3 s. The same image contrast settings were applied to each image. **e**–**h**, The bioluminescence signal from the same cells imaged in **a**–**d**. Exposure time, 0.3 s. Substrate, Nano-Glo Live Cell Substrate. The same image contrast settings were applied to each image. **i**, The change in bioluminescence intensity of a representative cluster of PINK1–HiBiT/LgBiT cells following CCCP injection. The black arrow indicates the time point of CCCP addition. Substrate, Nano-Glo Vivazine Substrate. **j**,**k**, Denoised images of HEK293T cells expressing PINK1–HiBiT/LgBiT without (**j**) and with (**k**) 10 μM CCCP treatment (5 h) measured on the EMCCD/LV200. Inset shows a zoomed-in image of one cell. Image contrast settings were adjusted to optimally show bioluminescence localization. Substrate, Nano-Glo Live Cell Substrate. **l**, The intensity profile of the dotted line in **k** is plotted. **m**,**n**, Denoised images of PINK1–HiBiT/LgBiT cells without (**m**) and with (**n**) 10 μM CCCP treatment (5 h) measured on the QIScope/6.5×. Inset shows a zoomed-in image of one cell with the same dimensions as the inset in **k**. Image contrast settings were adjusted to optimally show bioluminescence localization. Substrate, Nano-Glo Live Cell Substrate. **o**, Intensity profile of the dotted line in **n** is plotted. Effective pixel size of **a**–**h**, 423 nm. Effective pixel size of LV200/EMCCD, 800 nm. Effective pixel size of QIScope/6.5×, 169.2 nm. Representative results are shown from two to five independent experiments.

We first conducted a comparison of bioluminescence and fluorescence sensitivity by imaging MEFs expressing a fusion protein of Gamillus, a pH-insensitive green fluorescent protein^60^, and NLuc, driven by an inducible promoter. By adjusting concentrations of doxycycline hyclate (dox), different protein levels could be compared while ensuring a 1:1 reporter ratio. As expected, background autofluorescence from excitation light obscures the Gamillus signal at low protein expression levels (Fig. 5a–d). In contrast, the NLuc signal remains clear under the same expression levels (Fig. 5e–h; see also Supplementary Fig. 8 for a similar fusion protein expressed in HEK293T cells). These comparisons highlight the sensitivity of bioluminescence and hence, its utility for imaging low-abundance proteins.

**Extended Data Fig. 9:**
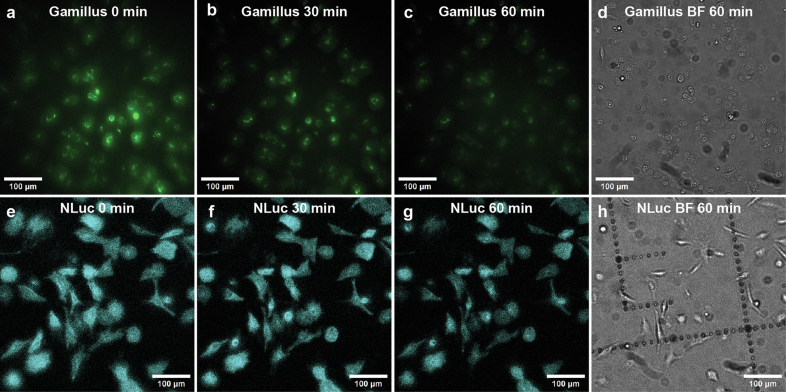
Toxicity and Bleaching Comparison of Fluorescence and Bioluminescence. **a–c**, Time-lapse images of the fluorescence of Gamillus-CD63 expressed in MEFs. embryonic. The same image contrast settings have been applied to each image. Exposure time: 3 ms. **d**, Transmission brightfield image shows that cell morphology changed significantly after 60 minutes of measurement. **e–g**, Time-lapse images show the bioluminescence of NLuc-CD63 MEFs. Exposure time: 0.3 s. Substrate: Nano-Glo Live Cell Substrate. **h**, Transmission brightfield image shows that cell morphology remained essentially unchanged after 60 min of measurement. Effective pixel size: 423 nm. Representative results are shown from three independent experiments.

Next, we assessed the impact of prolonged measurement on cells by continuously imaging MEFs overexpressing Gamillus–CD63 or NLuc–CD63. Over the course of 1 h, the fluorescence intensity of Gamillus–CD63 cells decreased gradually, accompanied by large-scale changes in cell morphology (Extended Data Fig. 9a–d and Supplementary Fig. 9), indicative of photobleaching and phototoxicity. In contrast, the bioluminescence intensity of NLuc–CD63 cells weakened slightly, without any observable change in cell morphology (Extended Data Fig. 9e–h). This comparison underscores the relatively high photostability and low toxicity of bioluminescence, which is advantageous for long-duration measurements.

These observations suggest that bioluminescence may be particularly suitable for extended-duration imaging of low-abundance proteins in live cells. To demonstrate this combined utility, we imaged PTEN-induced kinase 1 (PINK1), a key regulator of mitochondrial quality control. Under basal conditions, cellular PINK1 levels are extremely low due to continuous degradation following brief binding to mitochondria. However, in damaged mitochondria, PINK1 stabilizes on the outer mitochondrial membrane, selectively recruiting the ubiquitin ligase Parkin to initiate mitophagy^61^. Dysregulation of PINK1 signaling has been linked to neurodegenerative diseases such as Parkinson’s disease, autosomal recessive juvenile parkinsonism and amyotrophic lateral sclerosis, and is a topic of intense study^61,62^. However, the hours-long subcellular redistribution of PINK1 from low cytoplasmic levels to mitochondrial accumulation has been difficult to capture by fluorescence^61,63,64^.

**Extended Data Fig. 10:**
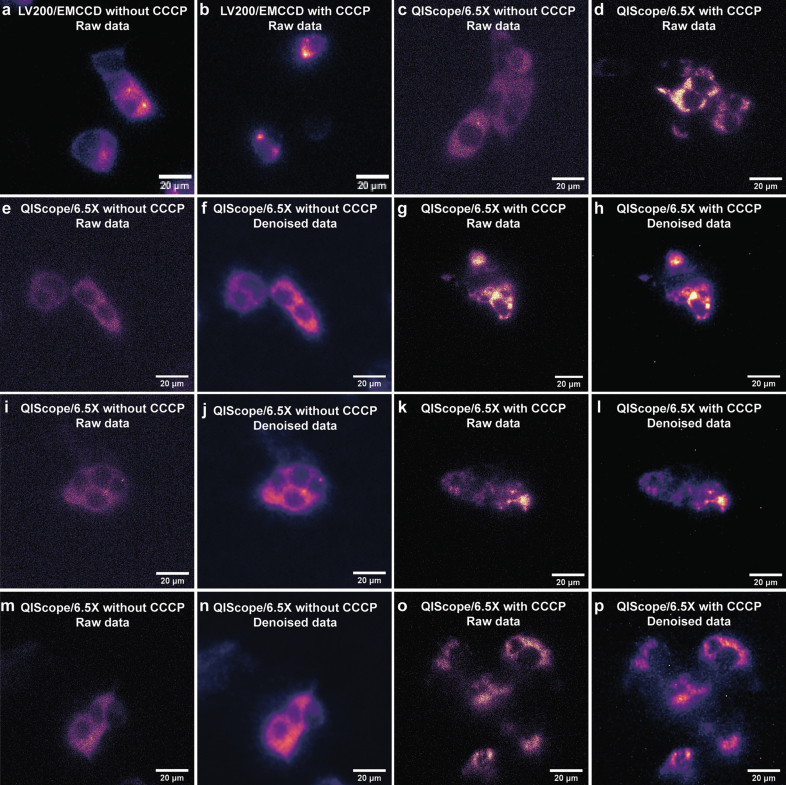
Imaging PINK1-HiBit/LgBiT using the LV200/EMCCD and QIScope/6.5×. **a, b**, Raw bioluminescence images of PINK1-HiBiT/LgBiT-expressing HEK293T cells without (**a**) and with (**b**) 10 μM CCCP (5 h) measured using the EMCCD/LV200. **c, d**, Raw bioluminescence images of PINK1-HiBit/LgBiT-expressing HEK293T cells with (**c**) and without (**d**) 10 μM CCCP (5 h) measured using the QIScope/6.5×. Exposure time: 50 s. Substrate: Nano-Glo Live Cell Substrate. **e**,**i**,**m**, Raw bioluminescence images of PINK1-HiBit/LgBiT-expressing HEK293T cells without CCCP measured using the QIScope/6.5×. Exposure time: 50 s. Substrate: Nano-Glo Live Cell Substrate. **f**,**j**,**n**, Denoised bioluminescence images of **e**,**i**,**m**, respectively. **g**,**k**,**o**, Raw bioluminescence images of PINK1-HiBit/LgBiT-expressing HEK293T cells with 10 μM CCCP (5 h) measured using the QIScope/6.5×. Exposure time: 50 s. Substrate: Nano-Glo Live Cell Substrate. **h**,**l**,**p**, Denoised bioluminescence images of **g**,**k**,**o**, respectively. Effective pixel size of LV200/EMCCD: 800 nm. Effective pixel size of Qiscope/6.5×: 169.2 nm. Representative results are shown from three independent experiments.

We imaged PINK1 tagged with HiBiT, a peptide that binds to the LgBiT protein coexpressed in the cytoplasm of HEK293T cells, to form a functional NLuc. We first monitored the increase in cellular bioluminescence intensity over several hours following addition of 10 μM carbonyl cyanide m-chlorophenyl hydrazone (CCCP), a mitochondrial uncoupler that triggers PINK1 translocation to mitochondria (Fig. 5i)^61,63^. Next, we compared images of PINK1 subcellular localization taken with the conventional LV200/EMCCD and a higher-resolution version of the QIScope (‘QIScope/6.5×’). Despite already possessing a 100× oil-immersion objective, the LV200/EMCCD was unable to clearly resolve changes in the spatial distribution of PINK1–HiBiT/LgBiT even with the aid of Noise2Info denoising (Fig. 5j–l and Extended Data Fig. 10a,b).

In contrast, the QIScope/6.5×, also equipped with a 100× oil-immersion objective and hence substantially higher spatial resolution, could clearly capture a change in subcellular localization of PINK1–HiBiT/LgBiT, consistent with translocation from the cytoplasm to mitochondria. Denoising resulted in a marked improvement in image quality (Fig. 5m–o and Extended Data Fig. 10c–p). The difference in spatial resolution between the two microscopy systems is particularly stark in intensity line profiles following CCCP treatment (Fig. 5l,o). Finally, with the detrimental effects of photobleaching or phototoxicity reduced in bioluminescence, PINK1–HiBiT/LgBiT dynamics could be continuously monitored for over 18 h (Supplementary Video 9).

## Discussion

Despite the advantages of bioluminescence, its use in live-cell imaging has been limited primarily by the performance of EMCCD cameras. In this study, we find that a QIS camera, developed by Gigajot Technology, outperforms the state-of-the-art EMCCD camera in direct benchmarking. Due to the small physical dimensions of the QIS sensor, we introduce a simple but unconventional microscope design, inspired by the Keplerian telescope, that maximizes signal detection using the QIS without sacrificing FOV.

The resulting ‘QIScope’ outperforms the state-of-the-art LV200/EMCCD bioluminescence microscope modestly in terms of SNR, but with substantially higher spatial image resolution, FOV and dynamic range. These capabilities enable static and dynamic live-cell imaging of low-abundance and high-abundance objects simultaneously, such as intracellular and extracellular vesicles or the protein PINK1—challenging experiments previously not possible by bioluminescence. Moreover, such measurements can be performed over long durations (>18 h) with minimal toxicity and probe bleaching. The simple and open design of the QIScope offers additional customizability, including straightforward integration of other imaging modalities such as epifluorescence.

The full range of capabilities for the QIScope has not been realized. The SNR could theoretically be boosted simply with brighter luciferase substrates such as fluorofurimazine or hikarazine-003 (ref. ^17^). These brighter substrates could then be used with different combinations of optics to obtain even greater spatial resolution (higher magnification) or greater SNR, depending on the application. The QIScope is currently optimized for blue-light detection, coinciding with the emission spectra of many bioluminescent reporters. However, wavelength multiplexing can be achieved with different enzyme–substrate pairs, along with spectral filter or phasor analysis^11,65^. The multi-modality of the QIScope can be readily expanded due to its open telescopic design. For instance, phase contrast or differential interference contrast imaging, previously not possible to integrate into conventional bioluminescence microscopes due to lack of space between the objective and tube lens, can now be incorporated. The telescopic setup also accommodates other small-sized detectors, such as single-photon avalanche diode arrays for time-of-flight imaging at the cellular level or next-generation QIS cameras, without sacrificing FOV.

The construction, performance and capabilities of the QIScope make bioluminescence an accessible and viable modality for live-cell imaging at high spatiotemporal resolution. All components of the QIScope were obtained commercially and can be integrated, or modified, in straightforward fashion. As reflected in our measurements of EVs, Gamillus–NLuc and PINK1, this system may be most applicable to challenging studies where high dynamic range, sensitivity, spatiotemporal resolution and long measurement durations are needed, particularly of photosensitive or autofluorescent samples that are unsuitable for fluorescence imaging. The enhanced FOV also permits measurements of larger samples such as organoids or tissues. Such studies would benefit not only from the growing array of bioluminescence reporters and sensors, but also from the modularity and multi-modality of the QIScope, allowing for bioluminescence integration with other analytical methods such as spatial proteomics or electron microscopy, with the familiar ease of fluorescence. In the toolbox of live-cell measurements, the QIScope expands the use cases of bioluminescence, offers facile mixing and matching with existing techniques and holds great potential as the basis of future technologies.

## Methods

### Benchmarking of sCMOS, EMCCD and QIS cameras

A home-built epifluorescence microscope was used to compare the sCMOS (Hamamatsu Fusion BT), EMCCD (Andor iXon 897) and QIS (Gigajot QIS16TS) cameras. A schematic is shown in Supplementary Fig. 1a. A 40× oil-immersion objective lens (UPLXAPO40XO, Olympus) and the tube lens of the LV200 microscope (Olympus) constitute the main body of the epifluorescence microscope. A supercontinuum white-light fiber laser (SuperK FIU-15, NKT Photonics) was used as the excitation source for fluorescence experiments. The SuperK VARIA filter system was used to select the center wavelength and bandwidth. The filtered light was sent to the microscope setup through the FD1 PM fiber and collimated (AC254–030-A-ML, Thorlabs).

The laser light was directed into the objective lens using a focusing lens (Thorlabs, AC254-045-A-ML) and reflected off of a beamsplitter (Thorlabs, BSN10R) to excite the sample. The collected fluorescence passed through the objective and beamsplitter, and a long-pass emission filter (Thorlabs, FELH0500) was used to reject laser light before it passed into the tube lens. To normalize the photon flux per pixel for the different cameras, ND filters were placed in front of the tube lens as follows: sCMOS, ND1.5 (NE10B-A and NE05B-A, Thorlabs); EMCCD, ND 2.4 (NE20B-A and NE04B-A, Thorlabs). No ND filters were used for the QIS. EM gain for the EMCCD was 300, a commonly used gain value selected as a compromise between sensitivity and dynamic range.

Using this microscope, a fluorescence microscope slide (Thorlabs, FSK4) was measured under 1 nW (average, $\sim 3\times {10}^{-7}{\rm{W}/{cm}}^{2}$; peak, $\sim 7\times {10}^{-5}{\rm{W}/{cm}}^{2}$) excitation intensity (460-nm wavelength, 5-nm bandwidth, 78.2-MHz repetition rate). The power was measured at the sample plane, and the laser spot size was determined from a fit to the beam profile at the sample using the 1/e² intensity values. For each measurement with the laser on, ten raw images were acquired (2-s integration time) and averaged together. The same steps were then taken with the laser off to obtain an image of the background. The images of the sample based on the three cameras were subtracted from the corresponding images of the background to obtain Fig. 1a–c. SNRs were calculated as follows: The signal was the maximum gray value of the ‘laser on’ images. The noise was obtained by taking the standard deviation of each ‘laser off’ image and then averaging them. These processing steps were performed in Fiji and Python.

Spontaneously immortalized MEFs labeled with colloidal quantum dots were used as a second sample for the camera benchmarking. MEFs were seeded on a glass coverslip (Marienfeld Superior Deckgläser, 12 mm) at 20,000–30,000 cells per coverslip. Cells were maintained for 24 h in ‘cell culture media’: DMEM (Thermo Fisher Scientific, 31053044) supplemented with 10% FBS (Gibco, 10270106), 5 mM sodium pyruvate (Sigma, S8636), 10 mM l-glutamine (Gibco, 25030024) and 0.5 mg ml^−1^ penicillin–streptomycin (Sigma P4333). The cells were cultured at 37 °C in a hypoxic workstation (DWS Whitley H35) with an atmosphere of 5% O_2_ and 7.5% CO_2_. After a 24-h incubation, the medium was removed and cells were fixed using 4% paraformaldehyde in H_2_O (1.1% dibasic sodium phosphate (Sigma, S51360100), 4% paraformaldehyde (Sigma, 158127) and 0.2% monobasic sodium phosphate (Sigma, S50110100) for 10 min at room temperature (RT).

The fixed cells were washed with Dulbecco’s phosphate-buffered saline (DPBS; Gibco, 14190136) for 10 min at RT. After washing, the fixed cells were blocked using ‘SUMI’ (2.5 mg ml^−1^ gelatin (Sigma, 104078) in a 1:1 dilution of Triton X-100, Sigma X100 and Tris-buffered saline; Sigma, 93350) for 45 min. Primary antibody against Acta2 (Rabbit polyclonal; Proteintech, 14395-I-AP) was added to the cells at a 1:1,000 dilution and incubated at 4 °C overnight. After overnight incubation, the coverslips were washed three times with DPBS for 10 min. After washing, the F(ab')_2_-Goat anti-Rabbit IgG (H + L) Secondary Antibody conjugated to Qdot 585 (Q-11411MP, Thermo Fisher) diluted in SUMI (1:50 dilution) was added to the cells and incubated at RT for 2 h. After incubation, the coverslips were washed three times with DPBS and mounted on a larger coverslip with Elvanol (Mowiol 0.4 g ml^−1^ (Sigma, 81381), 200 mM Tris at pH 8.5 (Sigma, T1819) and 30 mg ml^−1^ 1,4–diazabicyclo[2.2.2] octane; Sigma, D27802).

Labeled cells were imaged under 2.6-nW (average, $\sim 9\times {10}^{-7}{{\rm{W}/{cm}}}^{2}$; peak, $\sim 2\times {10}^{-4}{\rm{W}/{cm}}^{2}$) laser intensity (460-nm wavelength, 5-nm bandwidth, 78.2-MHz repetition rate) with a 500LP emission filter (FELH0500, Thorlabs) and 10-s integration time. The power was measured at the sample plane, and the laser spot size was determined from a fit to the beam profile at the sample using the 1/e² intensity values. Images were plotted in Fiji without additional processing. SNR values were calculated as described for the fluorescent slide. However, in this case, only one image file was used for the signal. EM gain for the EMCCD was 300.

For comparisons of the sCMOS, EMCCD and QIS cameras under the same effective pixel size, separate microscopes were built. Specifications of the cameras are found in Supplementary Table 1 and details of these setups are provided in Extended Data Fig. 1 and Supplementary Table 2.

The chip size comparison (Fig. 1g) was performed using the weak bioluminescence of the EXSISERS cells using the same optical setup, but with the EMCCD and QIS cameras exchanged. Exposure time was 100 s. For more details, see ‘EXSISERS cell line’.

### Construction of LV200/EMCCD and QIScope microscopes

Home-built LV200/EMCCD and QIScope microscopes were constructed for this study. The LV200/EMCCD (*M*_eff_ = 20×) consisted of a 100× oil-immersion objective lens (UPLXAPO100XO, Olympus), the LV200 tube lens (Olympus) and an EMCCD camera (iXon Ultra 897, Andor). The 100× oil-immersion objective was chosen to maximize light collection (NA = 1.45) and spatial image resolution.

The QIScope (*M*_eff_ = 2.6×) consisted of a 40× oil-immersion objective (UPLXAPO40XO, Olympus), a 20× air objective (LUCPLFLN20X, Olympus), two achromatic lenses in between (AC254-045-A-ML and AC254-035-A-ML, Thorlabs) and the QIS camera (Gigajot QIS16TS). The 40× oil-immersion objective was selected because it has a high NA (NA = 1.4) and relatively low magnification for an oil-immersion objective. The 20× air objective was selected for use as a tube lens because it gave a balanced combination of high magnification, long working distance and large back aperture.

For fluorescence imaging (Fig. 2h,i), a beamsplitter (BSN10R, Thorlabs) and 500LP emission filter (FELH0500, Thorlabs) were placed between the objective and the tube lens of the LV200/EMCCD (Supplementary Fig. 1b) and between the two lenses of the telescope of the QIScope (Fig. 2d). For transmission measurements, the beamsplitter and emission filters were removed for both setups.

From equation (1), equation (2) shows the photon flux per pixel is proportional to

2 $$\frac{\rm{photon}\; \rm{flux}}{\rm{pixel}}\propto \frac{{\rm{NA}}^{2}}{{M}_{\rm{eff}}^{2}}{d}^{2}$$

where *d* is the pixel size. From this, we find that, theoretically, the photon flux per pixel of the LV200/EMCCD setup is 3.8 times that of the QIScope under the same light emitted from a sample.

### Calculation of MTFs

The MTFs for the QIScope and LV200/EMCCD were calculated from transmission illumination images of a high-resolution microscopy test target (Edmund, TC-RT01) at a series of different spatial frequencies (*f*, parallel lines per mm). For each image, the illumination intensity was adjusted so that the maximum signal was approximately half the saturation intensity of the camera. Ten images were taken for each set of spatial frequencies and averaged together. For a given spatial frequency, a box was drawn over the set of five parallel lines and an average profile was obtained (Supplementary Fig. 2). The five peaks and four troughs were averaged together and used to calculate the contrast at each spatial frequency as $C=({I}_{\rm{{peak}}}-{I}_{\rm{{trough}}})/({I}_{\rm{{peak}}}+{I}_{\rm{{trough}}})$. The contrast as a function of spatial frequency, *C*(*f*), was normalized by the zero-frequency contrast, *C*(0), which was obtained from the maximum and minimum intensities in each image averaged over a selected area to give the MTF. That is, ${\rm{MTF}}(f)=C(f)/C(0)$. Each microscope’s MTF(*f*) was fitted with a cubic polynomial function, as guides for the eye, and the *x*-intercept gives the spatial resolution limit (Fig. 2g).

The theoretical ratio of the spatial resolution limits of the two microscopes can be calculated from the *M*_eff_ of each microscope and the pixel size (*d*) of their respective cameras as shown in equation (3):

3 $${\rm{Resolution}\;\rm{ratio}=\frac{{\it{M}}_{\rm{eff},\rm{QIScope}}/{\it{d}}_{\rm{QIS}}}{{\it{M}}_{\rm{eff},\rm{LV}200/\rm{EMCCD}}/{\it{d}}_{\rm{EMCCD}}}}$$

From this equation, we find that, theoretically, the QIScope has a spatial image resolution 1.89 times that of the LV200/EMCCD.

### Calculating SNRs for LV200/EMCCD and QIScope microscopes

The SNRs of Fig. 2h,i were calculated from measurements of a fluorescent slide (FSK4, Thorlabs) under 2-pW (average, $\sim 7\times {10}^{-9}{\rm{{W}/{cm}}}^{2}$; peak, $\sim 3\times {10}^{-6}\,{\rm{W/{cm}}}^{2}$) excitation from the SuperK (470-nm wavelength, 5-nm spectral bandwidth, 78.2-MHz repetition rate). The power was measured at the sample plane and the laser spot size was determined from a fit to the beam profile at the sample using the 1/e² intensity values. A 500-nm long-pass filter was placed in the emission path (FELH0500, Thorlabs). Schematics for the two setups are shown in Supplementary Fig. 1b and Fig. 2d. EM gain for the EMCCD was 300.

For each setup, the following steps were taken. Ten images were taken with the laser on and another ten with the laser off (background) with 10-s integration times. The respective signal and background images were averaged together, and the resulting background image was subtracted from its corresponding signal images to give the final image. Intensity profiles were obtained from a horizontal line across the center of each image and fitted with a Lorentzian function with baseline using a Python script. The peak value of the fitted function minus the baseline served as the signal intensity. The standard deviation of each background image was obtained and averaged together to give the final noise value. The ratio of the signal value to the noise value gave the final SNR.

For benchmarking the QIScope against the EMCCD at the same effective pixel size, separate optical setups were constructed. Details of these setups are provided in Extended Data Fig. 3 and Supplementary Table 4.

### EXSISERS cell line

HEK293T (ECACC, 12022001, Sigma-Aldrich) cells carrying the EXSISERS reporter system in the *MAPT* locus (NLuc luciferase in Exon 10)^40^ were maintained at 37 °C in a H_2_O-saturated atmosphere with 5% CO_2_. The cells were cultured in Advanced DMEM (Gibco, 12491015) supplemented with GlutaMAX (Gibco 31053044), 100 µg ml^−1^ penicillin–streptomycin and 10% FBS (Gibco, 10091148). At 90% confluency, cells were split by washing with DPBS and detached with Accutase (Gibco, A1110501) treatment for 10 min. Cells were then transferred into a new T75 flask at an appropriate density with fresh media or counted for seeding on eight-well chamber slides for microscopy (Grid-500 µ-Slides, Ibidi) for luminescence microscopy. Cells were plated 24 h before imaging at variable seeding density. The Nano-Glo Vivazine Substrate (Promega, N2580) was used according to the manufacturer’s protocol in a 1:100 final dilution, and imaging was performed 1.5 h after substrate addition.

For EXISISERS cell measurements (Extended Data Figs. 4 and 5), no excitation source was used, and filters and beam splitters were removed (Supplementary Fig. 1c). The exposure time was 10 s. Raw images were plotted in Fiji and presented without additional image processing. The SNRs were obtained by analyzing the cells and their surroundings in Fiji. EM gain for the EMCCD was 300.

### EV NLuc reporter cell lines

Spontaneously immortalized MEF cells^66^ expressing NLuc targeted to different subcellular localizations were generated from the cDNA of NLuc containing the high-affinity tag ALFA^67^ first cloned into a lentiviral expression plasmid (pLV-EF1a-IRES-Neo; Addgene, 85139). Plasma membrane targeting of NLuc was achieved by N-terminal addition of the myristoylation/palmitoylation sequence of the tyrosine kinase Lck to the NLuc sequence. The exosomal marker CD63 was N-terminally fused to the ALFA tagged NLuc sequence. DNA sequences can be found in the Supplementary Information.

Subsequently, HEK293T cells were transfected with a second-generation lentiviral packaging system (psPAX2; Addgene, 12260), together with the previously cloned reporter constructs, to produce lentiviral particles pseudotyped with the ecotropic envelope protein of moloney murine leukemia virus (pHCMV-EcoEnv; Addgene, 15802). MEF cells were infected with the lentiviral particles and selected with 750 µg ml^−1^ geneticin for 2 weeks to generate stable NLuc reporter MEFs. Protein expression in cells was verified by western blot (Supplementary Fig. 4) using a recombinant anti-ALFA single-domain antibody fused to a Guinea Pig IgG Fc domain (N1584, NanoTag Biotechnologies) and a Goat Anti-Guinea pig IgG H&L (HRP) secondary antibody (ab6908, Abcam; 1:10,000 dilution). Immunoblotting of valosin-containing protein was used as a loading control (2648S, Cell Signaling Technology). The presence of NLuc in EVs was confirmed by size-exclusion chromatography (Supplementary Fig. 5) with the qEVoriginal/35 nm Gen 2 column (Izon).

MEF cells expressing NLuc were seeded on microscopy chamber slides (µ-Slide VI 0.4 ibiTreat or µ-Slide 8 Well high Grid-500, ibidi) and incubated for 5 h in a regulated atmosphere of 20% O_2_ and 5% CO_2_ at 37 °C. After incubation, the cell culture medium was removed and the NLuc substrate was added. For Fig. 3 and Extended Data Figs. 6 and 8, the mixture of buffer and substrate from the Nano-Glo Live Cell Substrate (Promega, N205A) was added. The samples were measured within 5 min of addition. The exposure time of Cyto–NLuc and Myrplam–NLuc was 1 s and that of NLuc–CD63 was 2 s. For Extended Data Fig. 7, the Nano-Glo Vivazine Substrate (Promega) was used according to the manufacturer’s protocol in a 1:100 final dilution. Bioluminescence images were acquired after 1.5 h using 10-s exposure times. For bioluminescence measurements, the excitation laser was not used, and the beamsplitter and filters were removed from the setup. EM gain for the EMCCD was 300.

The dynamic range (Fig. 3d,h) was calculated as the ratio between the maximum and minimum gray values for their respective images (Fig. 3a,e). The maximum value was simply the maximum gray value of the entire image. The minimum value was the average of a small portion of the image in the extracellular space absent of any EV signal.

Single-particle imaging and tracking was performed on individual EV particles found in Supplementary Videos 5 and 6. Single-image frames of the particles were plotted in Fiji and shown in Extended Data Fig. 8c,e without additional image processing. Line profiles were plotted with fits to Gaussian functions in Python. Diffusion traces were obtained by manually determining the center coordinates of the particles in each frame using Fiji. The traces of these positions were plotted using Python in Extended Data Fig. 8d,f. The average two-dimensional diffusion velocity was calculated from averaging the physical distance traveled by each particle between frames and then dividing by the integration time (2 s). Gamma transformation was applied when displaying high-contrast images using Python (Fig. 3b,f and Extended Data Fig. 7d–f).

### Multimodal bioluminescence and epifluorescence imaging

MEFs expressing NLuc–CD63 were seeded on eight-well microscopy chamber slides (µ-Slide 8 Well high Grid-500, ibidi) and incubated in a regulated atmosphere of 20% O_2_ and 5% CO_2_ at 37 °C. After a 3-h incubation, the medium was removed and replaced with fresh DMEM containing ~50 nM MitoTracker Red CMXRos Dye. The dye was incubated for 30 min and then the medium was replaced with fresh DMEM. After another 30 min, the Nano-Glo Live Cell Substrate was added to the medium with a 50-fold dilution.

Bioluminescence images were acquired first with a 2-s integration time. The excitation light source was not used, and no emission filter was present in the setup. However, the beamsplitter was already in the setup (Fig. 2d). Before epifluorescence could be performed, a 600LP emission filter (FELH0600, Thorlabs) was manually inserted into the emission path. Epifluorescence imaging was performed under 1.63-μW (average, $\sim 2\times {10}^{-4}\,{\rm{W/{cm}}}^{2}$; peak, $\sim 0.2\,{\rm{W/{cm}}}^{2}$) excitation intensity (575-nm wavelength, 5-nm spectral bandwidth, 78.2-MHz repetition rate) and 0.5-s exposure time. The power was measured at the sample plane and the laser spot size was determined from a fit to the beam profile at the sample using the 1/e² intensity values. Raw data with pseudocoloring were plotted in Fiji and shown without additional image processing (Fig. 4).

### Gamillus–NLuc fusion cell line

A DNA sequence containing a double-reporter Gamillus–NLuc fusion and a high-affinity C-terminal ALFA tag was synthesized by Twist Bioscience and cloned into the self-inactivating lentiviral vector with inducible kinetics pSLIK-neo (25735, Addgene) using Gateway recombination cloning. To produce lentiviral particles, HEK293T cells were co-transfected with a second-generation lentiviral packaging system psPAX2 (Addgene, 12260) and envelope plasmid pHCMV-EcoEnv (Addgene, 15802) along with the pSLIK-Gamillus–NLuc, using PEI MAX (24765, Polysciences). After 72 h, the supernatant containing lentiviral particles was isolated and filtered from the cell debris. MEFs were infected and incubated overnight in the medium supplemented with 10 µg ml^−1^ protamine. Subsequently, cells were selected with 750 µg ml^−1^ geneticin (2039.2, Roth) for 2 weeks to generate a stable MEF line expressing Gamillus–NLuc. To verify the expression of the doxycycline-inducible Gamillus–NLuc, cells were treated with different concentrations of dox (2–25 µg ml^−1^; D9891, Sigma-Aldrich). The Gamillus signal was validated by fluorescence microscopy and the protein expression by immunoblot using a recombinant anti-ALFA single-domain antibody fused to a Guinea Pig IgG Fc domain (N1584, NanoTag Biotechnologies) and a Goat Anti-Guinea pig IgG H&L (HRP) secondary antibody (ab6908, Abcam), with a valosin-containing protein as a loading control (2648S, Cell Signaling Technology). DNA sequences can be found in the Supplementary Note 1.

MEFs expressing Gamillus–NLuc were seeded on microscopy chamber slides (µ-Slide 8 Well high Grid-500, ibidi) and incubated for 24 h with different dox concentrations in a regulated atmosphere of 20% O_2_ and 5% CO_2_ at 37 °C. To avoid the impact of bioluminescence on fluorescence, fluorescence experiments were first performed. Before epifluorescence could be performed, a GFP Emission Filter (MF525-39, Thorlabs) was manually inserted into the emission path. Epifluorescence imaging was performed with samples in a stage-top incubator (H301-K-FRAME, OkoLab) under 40-μW (average, $\sim 5\times {10}^{-3}{{\rm{W}/{cm}}}^{2}$; peak, $\sim 2\,{\rm{W/{cm}}}^{2}$) excitation intensity (470-nm wavelength, 20-nm spectral bandwidth, 78.2-MHz repetition rate) and 0.3-s exposure time. The power was measured at the sample plane, and the laser spot size was determined from a fit to the beam profile at the sample using the 1/e² intensity values. Raw data with pseudocoloring were plotted in Fiji and shown without additional image processing (Fig. 5a–d). For Fig. 5e–h, the Nano-Glo Live Cell Substrate (Promega) was used according to the manufacturer’s protocol in a 1:100 final dilution. Bioluminescence images were acquired using 0.3-s exposure times. For bioluminescence measurements, the excitation laser was not used, and the beamsplitter and filters were removed from the setup.

### NLuc–msfGFP fusion cell line

We transfected HEK293T cells (ECACC, 12022001) with a hyperactive PiggyBac transposase plasmid combined with a transposon plasmid encoding a TRE3g-driven NanoLuc-luciferase–msfGFP fusion. The transposon also encodes CAG-driven blasticidin deaminase coupled to TetON3g transactivator expression via P2A. Cells were transfected in six-well format. After expansion to a T75 flask, cells were selected with 3 µg ml^−1^ blasticidin followed by FACS sorting to obtain a population with low basal expression without addition of doxycycline. The procedures for seeding, imaging and data processing of HEK293T cells expressing NLuc–msfGFP were identical to those used for Gamillus–NLuc.

### Phototoxicity/photostability measurements

MEF cells expressing Gamillus–CD63 were produced in the same manner as MEFs expressing NLuc–CD63. MEF cells expressing Gamillus–CD63 and NLuc–CD63 were seeded on microscopy chamber slides (µ-Slide 8 Well high Grid-500, ibidi) and incubated for 3 h in a regulated atmosphere of 20% O_2_ and 5% CO_2_ at 37 °C. Gamillus–CD63 in MEF cells were imaged under 2.54-mW laser intensity (average, $\sim 0.5\,{\rm{W/{cm}}}^{2}$; peak, $\sim 175\,{\rm{W/{cm}}}^{2}$, 460-nm wavelength, 70-nm bandwidth, 78.2-MHz repetition rate) with a GFP Emission Filter (MF525-39, Thorlabs) and 3-ms integration time. The power was measured at the sample plane and the laser spot size was determined from a fit to the beam profile at the sample using the 1/e² intensity values. Extended Data Fig. 9a–d and Supplementary Fig. 9 were plotted in Fiji without additional processing. Gamillus–CD63 in MEF cells were placed in a home-built flow system integrated with the stage-top incubator using a mixture of Nano-Glo Live Cell Substrate (Promega) in cell culture media (1:100 dilution) at a flow rate of 100 μL min^−1^. Bioluminescence images were acquired using 0.3-s exposure times. For bioluminescence measurements, the excitation laser was not used, and the beamsplitter and filters were removed from the setup. Extended Data Fig. 9e–h and Supplementary Fig. 9 were also plotted in Fiji without additional processing.

### PINK1–HiBiT/LgBiT cell line

HEK293T cells (ECACC, 12022001) were transfected with a hyperactive piggyBac transposase^68,69^ combined with a transposon plasmid encoding TRE3g-driven PINK1 with a HiBiT tag (Promega) fused to the C terminus. Additionally, the transposon comprises a CAG-promoter-driven LgBiT, which complements HiBiT to form a functional NLuc reporter (HiBiT/LgBiT) tethered to PINK1. To enable positive selection of the engineered cells, the CDS of the puromycin *N*-acetyltransferase was cloned together with an upstream IRES downstream of the LgBiT CDS. Also, the transposon included a PGK1-promoter-driven TetON3G transactivator to drive PINK1–HiBiT expression upon addition of low levels of doxycycline. Briefly, to generate the cell line, 25,000 cells were seeded in 96-well format and transfected after 24 h. Two to three days following transfection, cells were expanded to a six-well plate followed by puromycin selection (1 µg ml^−1^). After 2 weeks, cells were monoclonalized by limited dilution followed by functional validation of the respective clones via doxycycline induction.

Bioluminescence imaging of PINK1–HiBiT/LgBiT cells was performed as follows. First, cells were plated ~24 h before imaging at variable seeding density with 5 ng ml^−1^ dox in the incubator. For tracking PINK1 dynamics, the Nano-Glo Vivazine Substrate (Promega) was used according to the manufacturer’s protocol in a 1:100 final dilution, and imaging was performed 1.5 h after substrate addition in the stage-top incubator. After collecting the first image, 10 μM CCCP was added by replacing two-thirds of the existing growth medium with fresh medium while maintaining the substrate concentration, and the remaining images were collected on the QIScope using 10 s exposure time (Fig. 5i). As a control, only cell culture medium was added. In Supplementary Video 9, 100 ng ml^−1^ dox was used with the QIScope/6.5×. Exposure time was 50 s.

For comparative imaging of PINK1–HiBiT/LgBiT subcellular localization using the LV200/EMCCD (Extended Data Fig. 10a,b and Fig. 5j,k) and QIScope/6.5× (Extended Data Fig. 10c,d and Fig. 5m,n), imaging was performed 5 h after 10 μM CCCP addition in the stage-top incubator. The Nano-Glo Live Cell Substrate (Promega) was used according to the manufacturer’s protocol in a 1:100 final dilution. Exposure time was 50 s.

### Image denoising

Image denoising was performed using two deep-learning methods based on the U-Net architecture. For single-frame data (Fig. 5j,k,m,n and Extended Data Fig. 7g–l), we used the Noise2Info approach^48^, a self-supervised technique that reconstructs each pixel of a noisy image using information from neighboring pixels and the pixel itself. This method estimates the upper bound of the noise standard deviation to weigh the information effectively. We followed the original model and hyperparameters, training on 64 × 64-pixel patches over 50,000 steps, updating the noise estimate every 1,000 steps.

For multi-frame time-lapse data (Fig. 3c,g and Supplementary Videos 1–8), we used the Noise2Noise approach^49^, generating semi-noisy targets by averaging the pixel-wise mean of seven adjacent frames, with the target frame at the center (Supplementary Fig. 10). This averaging reduces background noise while preserving cellular structure signals. We trained separate networks for each recording to handle specific nuances, maintaining consistent model architecture and hyperparameters. Each model was trained on 256 × 256-pixel crops with a batch size of 16 for 50 epochs, using a learning rate of 1e−4. Despite the high noise in PINK1 data (Supplementary Video 9), we opted for a Noise2Info model, as multi-frame averaging failed to produce viable targets for supervised training, following the same settings as for single-frame data.

Intensity gray values from the QIS16TS have been obtained in photon number resolving mode and should, in principle, represent detected photon numbers for raw (non-denoised) data. In denoised images, the intensity values can no longer be considered the number of detected photons.

### Materials availability

Materials related to the EXSISERS, PINK1 and NLuc–msfGFP cell lines can be obtained from G.W. and D.-J.J.T. Materials related to the EV cell lines, as well as the Gamillus–NLuc cell line, can be obtained from S.D.

### Reporting summary

Further information on research design is available in the Nature Portfolio Reporting Summary linked to this article.

## Online content

Any methods, additional references, Nature Portfolio reporting summaries, source data, extended data, supplementary information, acknowledgements, peer review information; details of author contributions and competing interests; and statements of data and code availability are available at 10.1038/s41592-025-02694-3.

## Supplementary information

Supplementary Information Supplementary Figs. 1–11, Supplementary Tables 1–4 and Supplementary Note 1.

Reporting Summary

Supplementary Video 1 Bioluminescence from MEFs expressing NLuc–CD63 acquired with the LV200/EMCCD (denoised). Exposure time, 2 s. Substrate, Nano-Glo Live Cell Substrate. See also Fig. 3a–c.

Supplementary Video 2 Bioluminescence from MEFs expressing NLuc–CD63 acquired with the QIScope (denoised). Boxes indicate regions containing diffusing particles. Substrate, Nano-Glo Live Cell Substrate. See also Fig. 3e–g and Extended Data Fig. 8b.

Supplementary Video 3 Bioluminescence from a Myrpalm–NLuc cell measured on the QIScope shown under low image contrast settings (denoised). The arrow points to movement of the plasma membrane indicative of macropinocytosis/endocytosis. Exposure time, 20 s. Substrate, Nano-Glo Vivazine Substrate. See also Extended Data Fig. 7b,h.

Supplementary Video 4 Bioluminescence from a NLuc–CD63 cell measured on the QIScope shown under low image contrast settings (denoised). Small puncta, likely MVBs, can be seen undergoing directed movement. Exposure time, 20 s. Substrate, Nano-Glo Vivazine Substrate. See also Extended Data Fig. 7c, i.

Supplementary Video 5 Bioluminescence from Myrpalm–NLuc cells measured on the QIScope shown under high image contrast settings (denoised). A diffusing particle, presumably an EV, can be seen between the cells. Exposure time, 2 s. Substrate, Nano-Glo Live Cell Substrate. See also Extended Data Fig. 8a,c,d.

Supplementary Video 6 Bioluminescence from NLuc–CD63 cells measured on the QIScope shown under high image contrast settings (denoised). A diffusing particle, presumably an EV, can be seen between the cells. Exposure time, 2 s. Substrate, Nano-Glo Live Cell Substrate. See also Extended Data Fig. 8b,e,f.

Supplementary Video 7 Bioluminescence from a Myrpalm–NLuc cell measured on the QIScope shown under high image contrast settings (denoised). The arrow points to the cell’s membrane extension moving and attaching to a surface-bound EV. Exposure time, 20 s. Substrate, Nano-Glo Vivazine Substrate.

Supplementary Video 8 Bioluminescence from a NLuc–CD63 cell measured on the QIScope shown under high image contrast settings (denoised). The arrow points to the transfer of a CD63-labeled cargo between two cells through a cellular tunneling nanotube. Exposure time, 2 s. Substrate, Nano-Glo Live Cell Substrate.

Supplementary Video 9 Bioluminescence from PINK1–HiBiT/LgBiT-expressing cells measured on the QIScope/6.5× (denoised). 10 μM CCCP was added to cells in the second frame. Exposure time, 50 s. Substrate, Nano-Glo Vivazine Substrate.

## Data Availability

Raw data for main text figures and Extended Data figures is publicly available on Zenodo via 10.5281/zenodo.14726231 (ref. ^70^).

## Code availability

Code used to analyze data is available under the MIT license at https://github.com/RuyuMa/A-Telescopic-Microscope-Equipped-with-a-Quanta-Image-Sensor-for-Live-Cell-Bioluminescence-Imaging.git/.
