# Supplementary material for: A telescopic microscope equipped with a quanta image sensor for live-cell bioluminescence imaging
Source: Nat Methods. 2025 May 29;22(6):1321–30. doi: 10.1038/s41592-025-02694-3 (PMC12165864; doi:10.1038/s41592-025-02694-3)
Supplement: Supplementary file 1 — Supplementary Figs. 1–11, Supplementary Tables 1–4 and Supplementary Note 1. [file 41592_2025_2694_MOESM1_ESM.pdf]

nature methods

Supplementary information https://doi.org/10.1038/s41592-025-02694-3

A telescopic microscope equipped with a quanta image sensor for live-cell bioluminescence imaging

In the format provided by the authors and unedited

Contents

Supplementary Fig. 1 | Different microscope setups using the LV200 tube lens.............................. 3

Supplementary Fig. 2 | Measuring the contrast for the modulation transfer function (MTF)............4

Supplementary Fig. 3 | Dynamic range comparison of the LV200/EMCCD and QIScope..................5

Supplementary Fig. 4 | Western blot of nLuc expression in mouse embryonic fibroblasts (MEFs). 6

Supplementary Fig. 5 | Size-exclusion chromatography of supernatants collected from NLuc-expressing mouse embryonic fibroblasts.. 7

Supplementary Fig. 6 | Background in bioluminescence imaging of NLuc MEFs and relation to secreted protein..8

Supplementary Fig. 7 | Bioluminescence imaging of EXSISERS cells with long exposure time...... 9

Supplementary Fig. 8 | Comparison of bioluminescence and fluorescence from a NLuc-msfGFP fusion expressed in HEK293T cells..10

Supplementary Fig. 10 Schematic of denoising multi-frame time-lapse data implemented with U-Net on QIScope...12

Supplementary Fig. 11 | Simulation of Hamamatsu Fusion BT (sCMOS), ORCA-Quest, and Gigajot QIS16TS using the Hamamatsu Camera Simulation Engine..13

Supplementary Table 1 | Comparison of Camera Specifications for the Hamamatsu Fusion BT, Andor iXon 897, Hamamatsu ORCA-Quest qCMOS, and Gigajot QIS16TS...................................... 14

Supplementary Table 2 | Specifications for setups built for comparing cameras at the same effective pixel size...15

Supplementary Table 3 | Signal-to-noise ratios obtained for camera comparisons using ND filters and same effective pixel size... 16

Supplementary Table 4 | Details of setups built for comparing the EMCCD and QIScope at the same effective pixel size...17

Supplementary Note..18

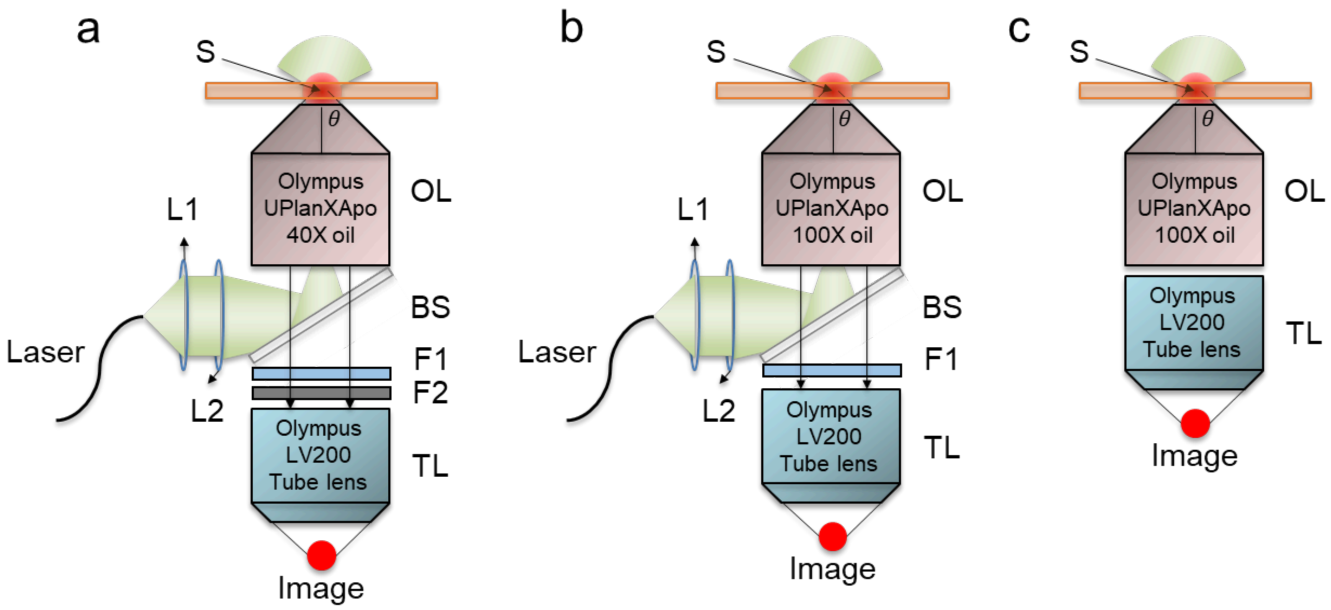

Supplementary Fig. 1 | Different microscope setups using the LV200 tube lens.

a, Schematic of microscope used for camera benchmarking (Fig. **1**). Distance between OL and TL: ~30 mm. **b,** LV200/EMCCD setup used in Fig. **2b** and **h**. Distance between OL and TL: ~30 mm. **c,** LV200/EMCCD setup used in Fig. **2e**, Fig. **3,** Extended Data Fig. **4,** Extended Data Fig. **5** and Extended Data Fig. **7**. Distance between OL and TL: ~0 mm. For all setups: L1: Lens 1, L2: Lens 2, S: Sample, OL: Objective lens, BS: Beamsplitter (BSN10R, Thorlabs), F1: Longpass Filter, F2: Neutral Density Filter, TL: Tube lens.

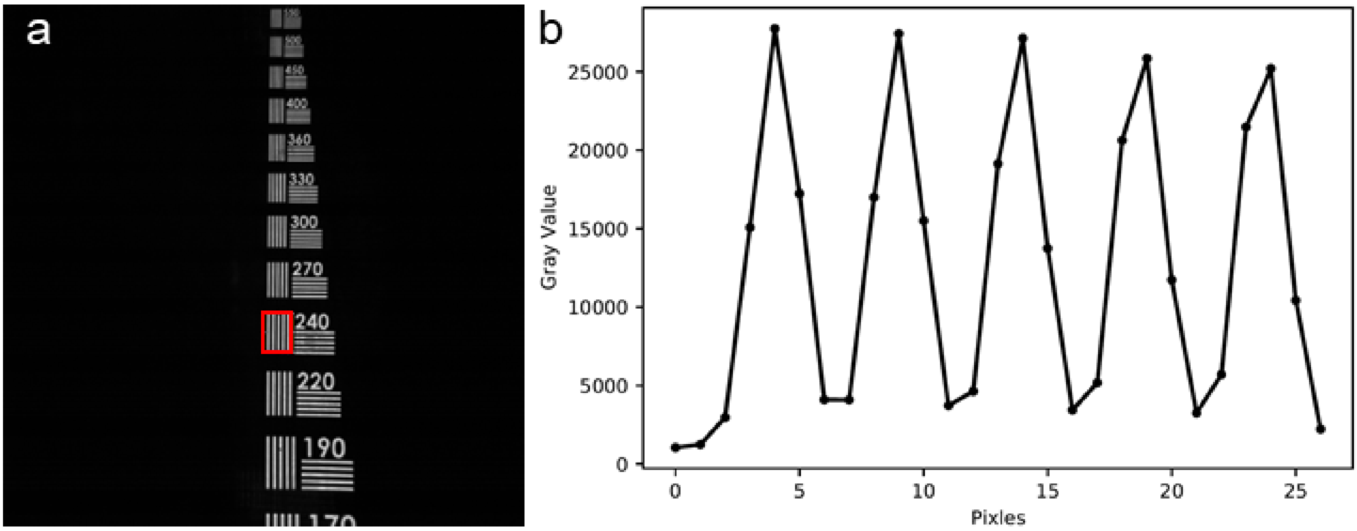

Supplementary Fig. 2 | Measuring the contrast for the modulation transfer function (MTF).

a, Example image of resolution test target taken on the LV200/EMCCD microscope. **b,** Profile of pixel grey values averaged over the red box in **a**. The contrast at a given spatial resolution was calculated from the averages of the peak and trough intensities of this profile.

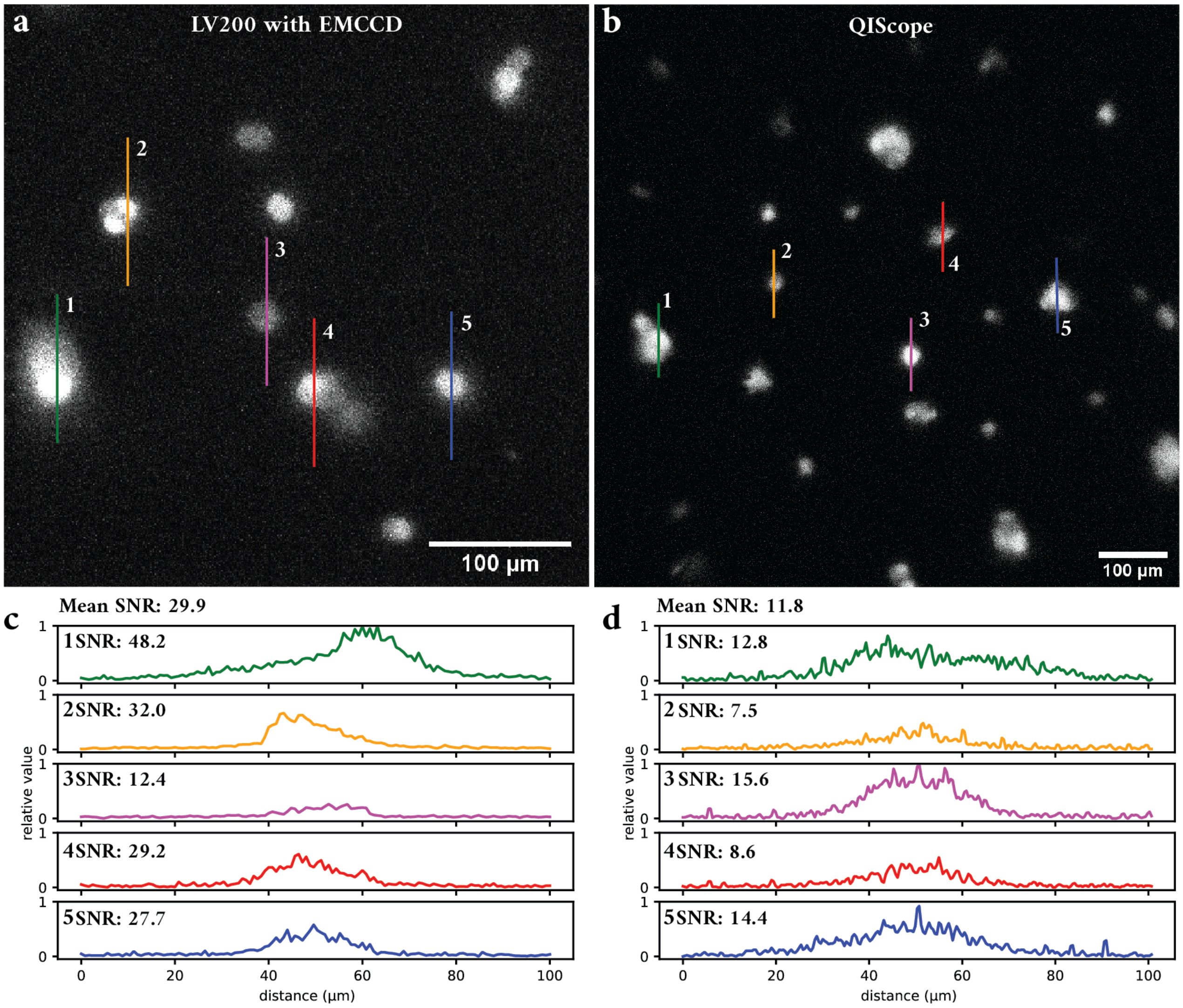

Supplementary Fig. 3 | Bioluminescence imaging of EXSISERS cells with long exposure time.

a,b, Images of bioluminescence from EXSISERS cells obtained by the LV200/EMCCD (**a**) and QIScope (**b**) microscopes using 100 s exposure time. Substrate: Nano-Glo Vivazine Substrate. **c,d,** Intensity line profiles over cells marked in **a,b** are plotted for the LV200/EMCCD (**c**) and QIScope (**d**), respectively. Peak SNR values are given for each trace, along with an average SNR for all five traces. The QIScope underperforms at very long integration times. Effective pixel size of LV200/EMCCD: 800 nm. Effective pixel size of Qiscope: 423 nm. Representative results are shown from three independent experiments.

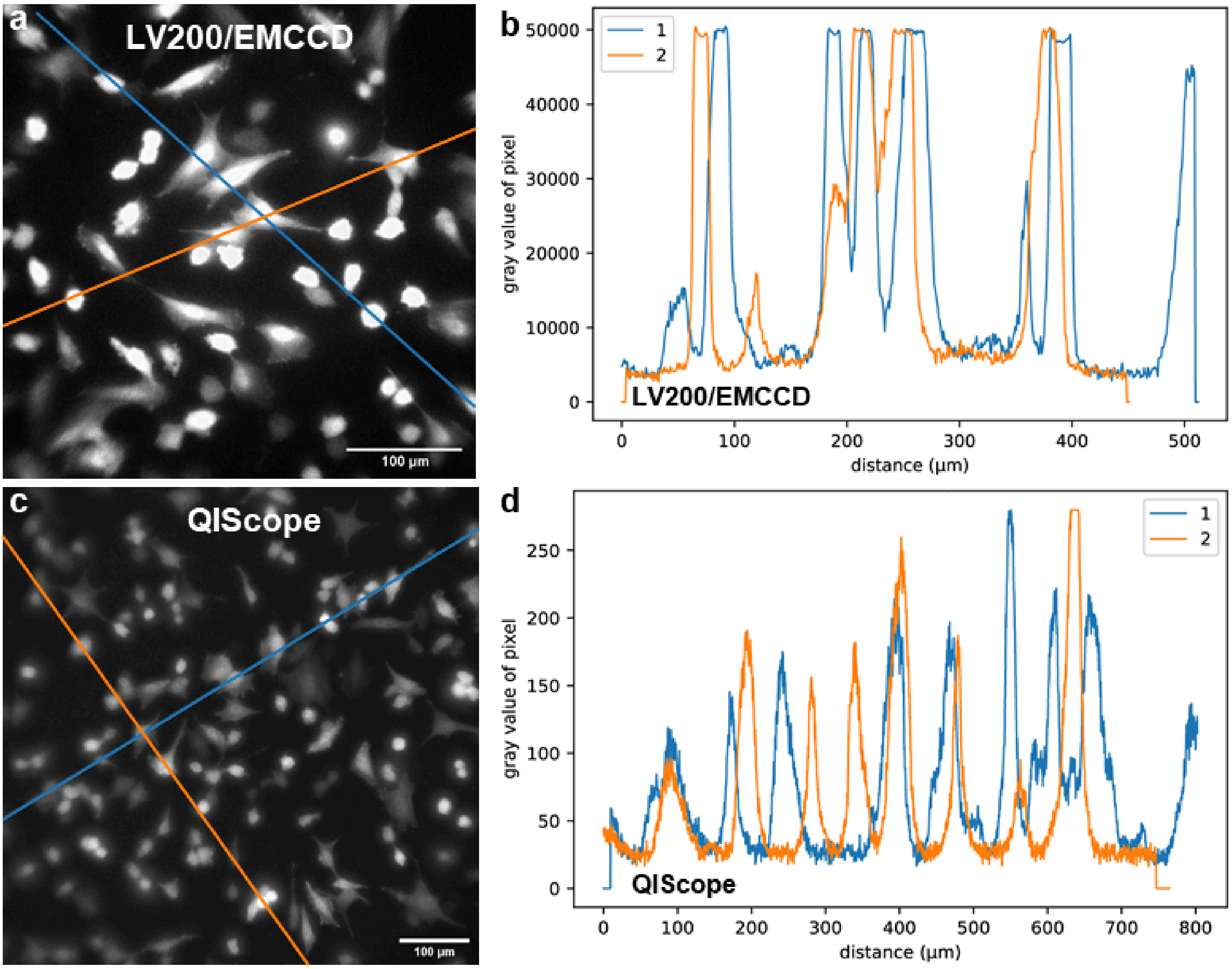

Supplementary Fig. 4 | Dynamic range comparison of the LV200/EMCCD and QIScope.

a,c, Bioluminescence images of Cyto-nLuc cells taken by the LV200/EMCCD and QIScope microscopes, respectively. Exposure time: 1s. Substrate: Nano-Glo Live Cell Substrate. **b,d,** Intensity line profiles marked in **a** and **c**, respectively. Many cells appear saturated on the LV200/EMCCD. Effective pixel size of LV200/EMCCD: 800 nm. Effective pixel size of Qiscope: 423 nm. Representative results are shown from two independent experiments.

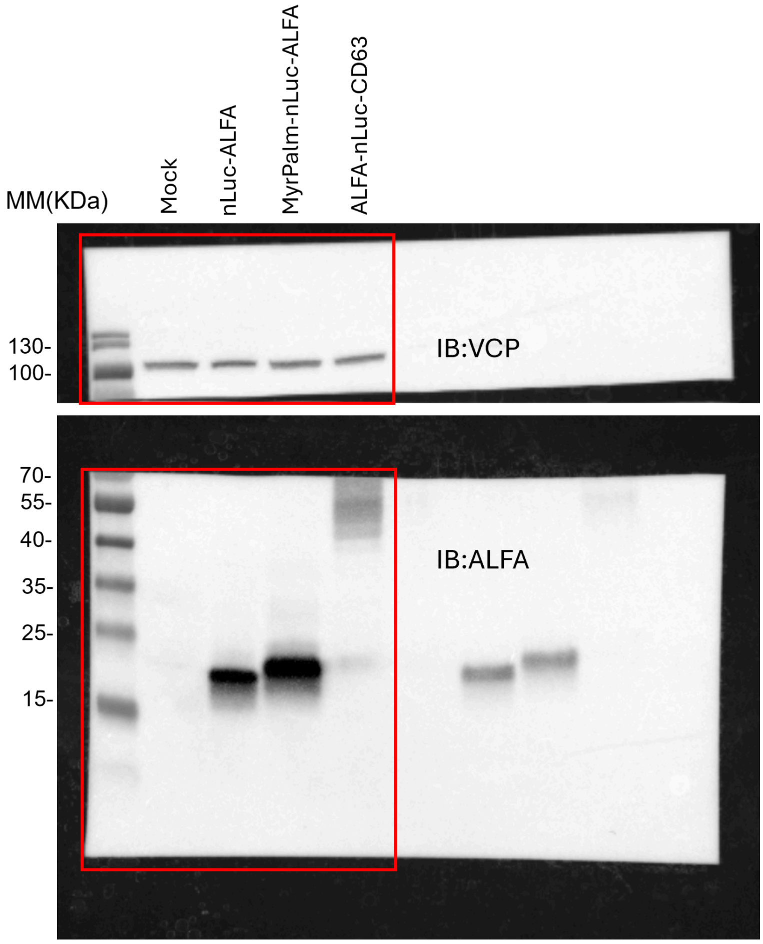

Supplementary Fig. 5 | Western blot of nLuc expression in mouse embryonic fibroblasts (MEFs). ALFA-tag immunoblotting shows no signal in control MEFs ("Mock"), lowest molecular weight band in Cyto-NLuc cells ("nLuc-ALFA"), slightly higher molecular weight band in MyrPalm-nLuc cells ("MyrPalm-NLUC-ALFA"), and higher molecular weight band in NLuc-CD63 cells ("ALFA-nLUC-CD63"). Valosin-containing protein (VCP) was used as a loading control.

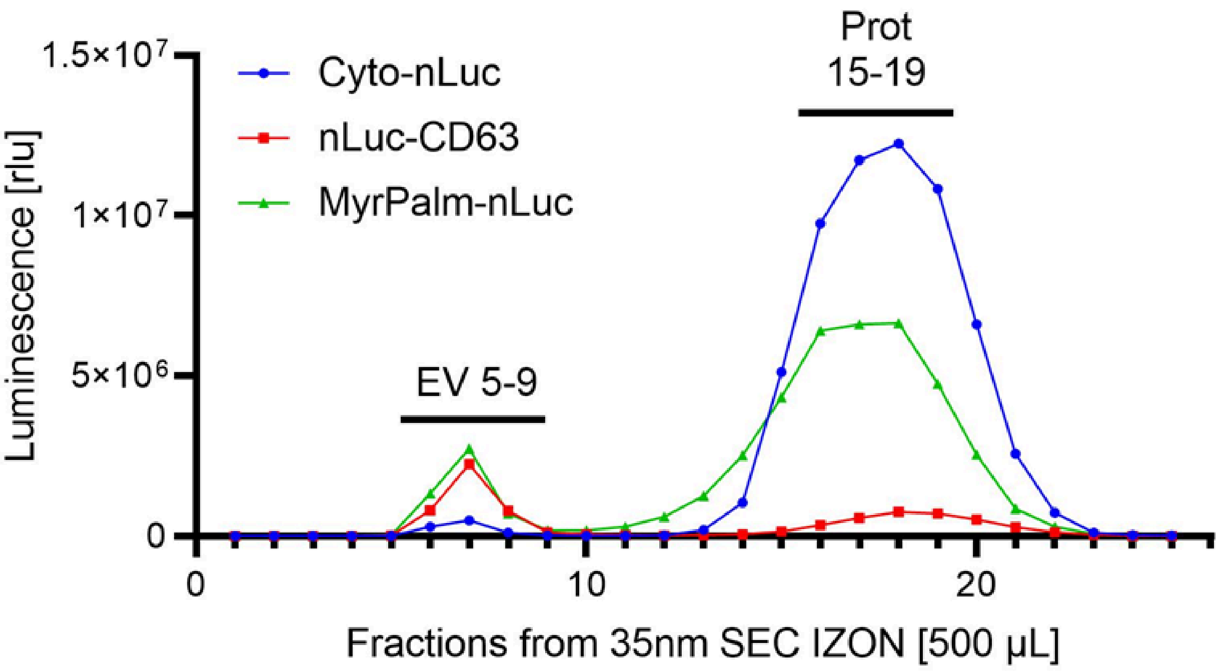

Supplementary Fig. 6 | Size-exclusion chromatography of supernatants collected from NLuc-expressing mouse embryonic fibroblasts.

The bioluminescence signal is shown as a function of elution fraction. The first elution peak corresponds to extracellular vesicles and the second elution peak corresponds to secreted protein.

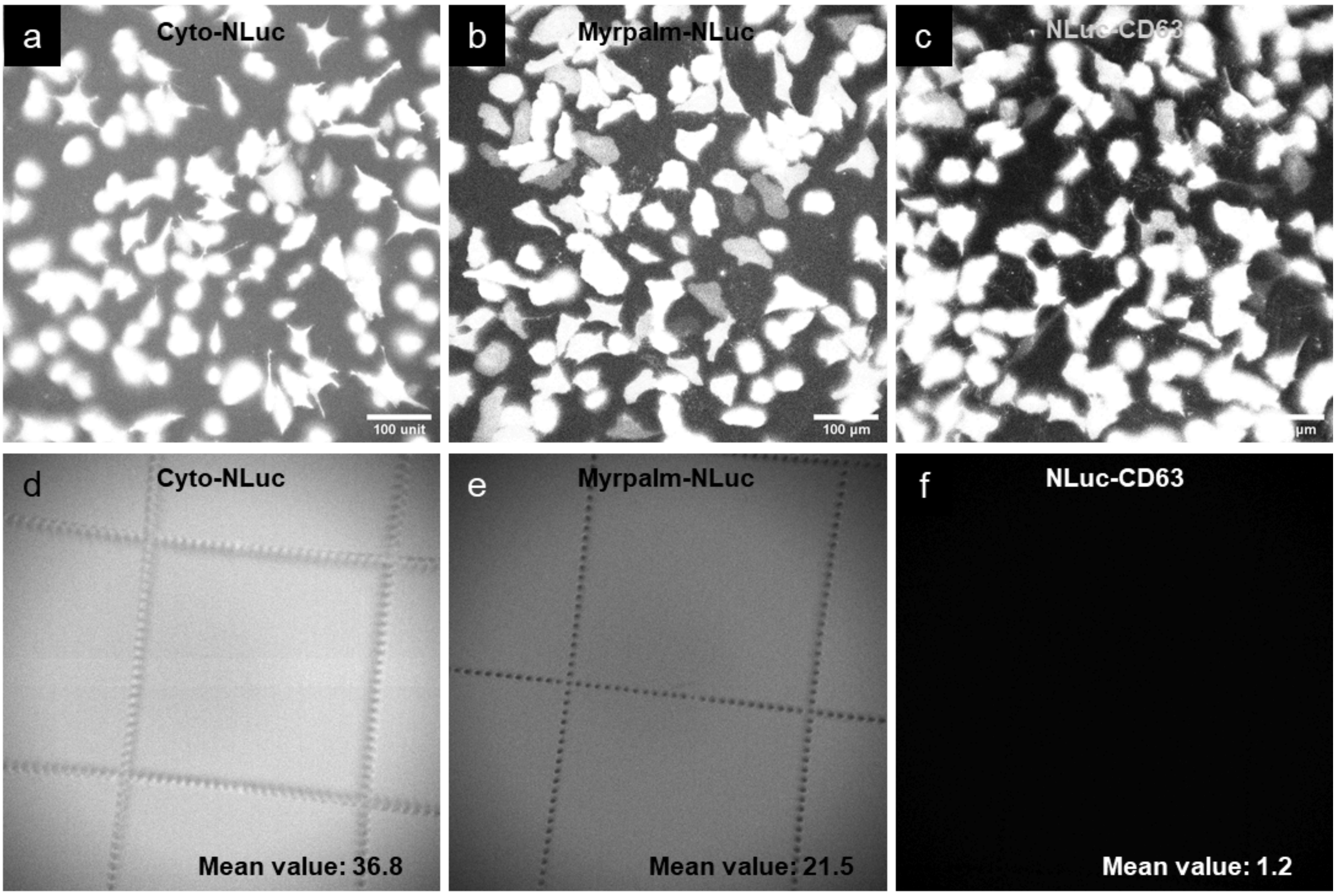

Supplementary Fig. 7 | Background in bioluminescence imaging of NLuc MEFs and relation to secreted protein.

a–c, Images of Cyto-NLuc (**a**), Myrpalm-NLuc (**b**), and NLuc-CD63 cells (**c**), respectively, using the Nano-Glo Live Cell Substrate and the QIScope. The contrast in (**b**) and (**c**) are the same, but the contrast in (**a**) is slightly lower due to the high background. **d–e**, The protein fractions from size exclusion chromatography (Fractions 15–19, **Supplementary Fig. 5**) obtained from the supernatants of Cyto-NLuc (**d**), Myrpalm-NLuc (**e**), and NLuc-CD63 cells (**f**) imaged on the QIScope with the Nano-Glo Luciferase Assay Substrate. Image contrast settings are identical. Effective pixel size: 423 nm. Representative results are shown from three independent experiments.

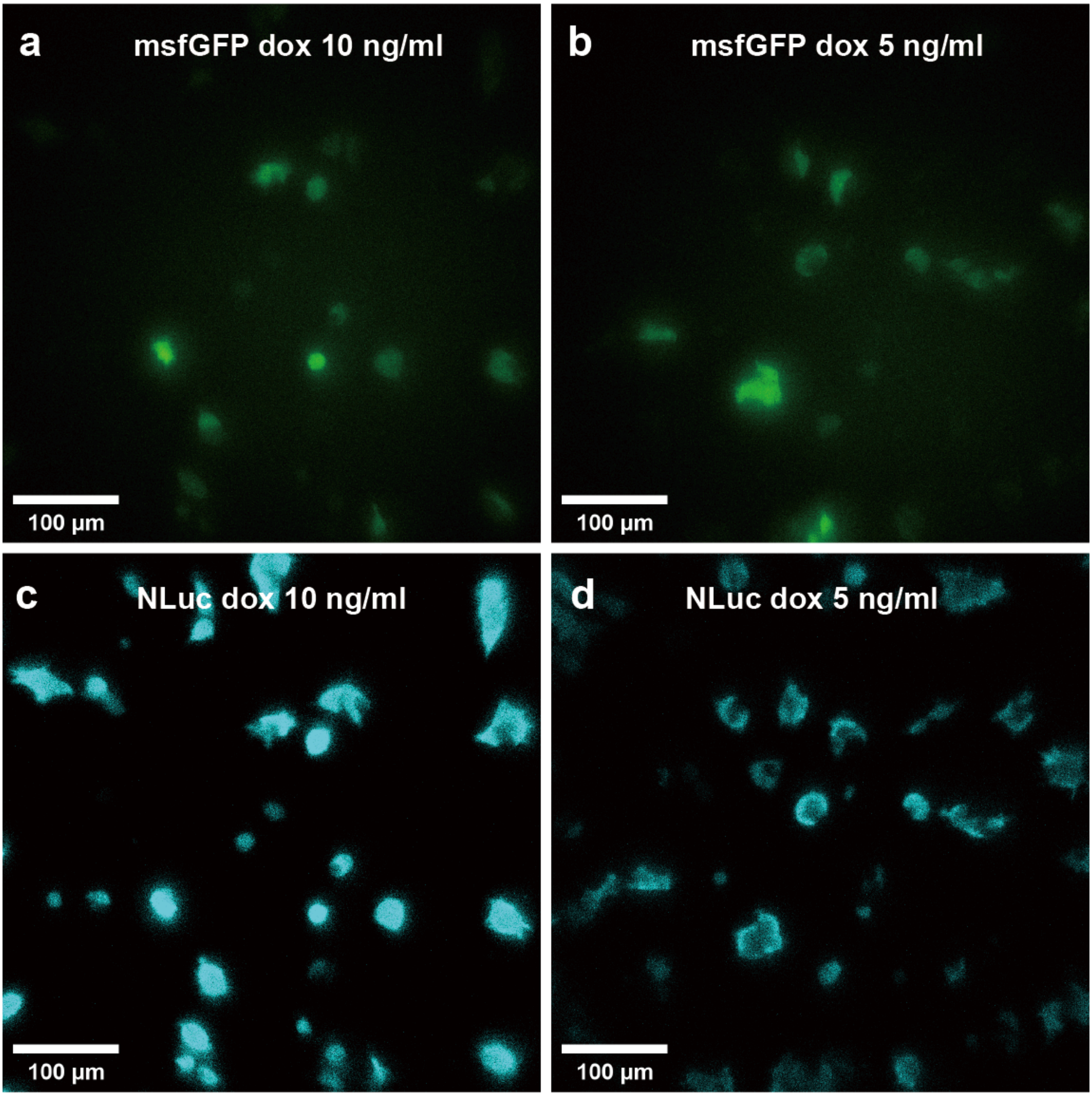

Supplementary Fig. 8 | Comparison of bioluminescence and fluorescence from a NLuc-msfGFP fusion expressed in HEK293T cells.

a,b, The fluorescence signal from HEK293T cells expressing a NLuc-msfGFP fusion protein imaged at different doxycycline hyclate (“dox”) levels. Exposure time: 0.3 s. The same image contrast settings were applied to each image. **c,d,** The bioluminescence signal from the same cells imaged in **a,b,** respectively. Exposure time: 0.3 s. Substrate: Nano-Glo Live Cell Substrate. The same image contrast settings were applied to each image. Representative results are shown from three independent experiments.

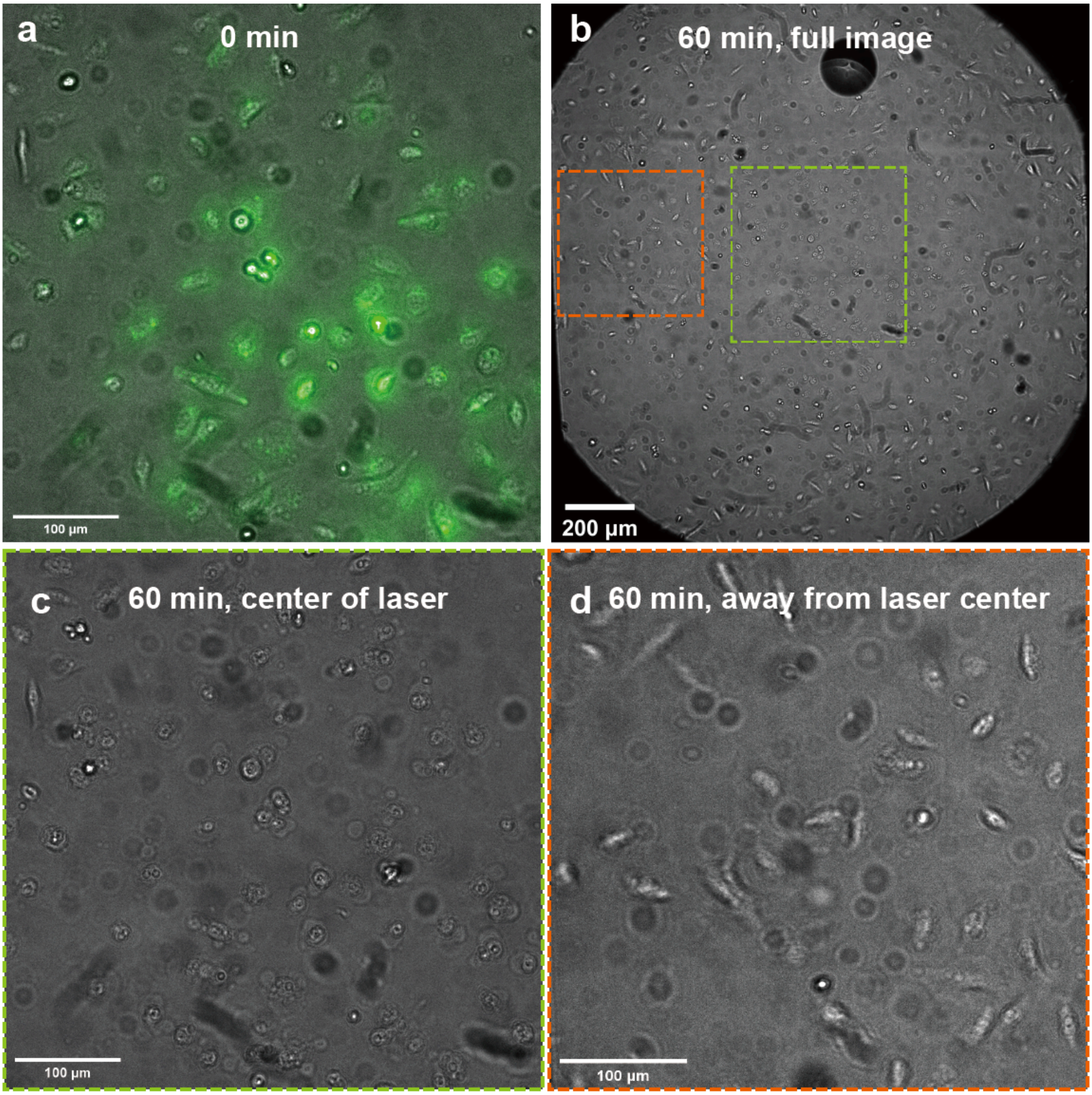

Supplementary Fig. 9 | Comparison of cell morphology before and after 60 min fluorescence illumination.

a, Transmission bright field (BF) image with fluorescence overlay showing cell morphology at 0 min. Fluorescence image here is the same data as in **Extended Data Fig. 9**. **b,** Full BF image of cells after 60 min laser exposure. **c,** Zoom-in of green dashed box in b showing cell morphology after 60 min exposure at the center of the laser spot. **d,** Zoom-in of orange dashed box in b showing cell morphology away from the center of the laser spot. Representative results are shown from three independent experiments.

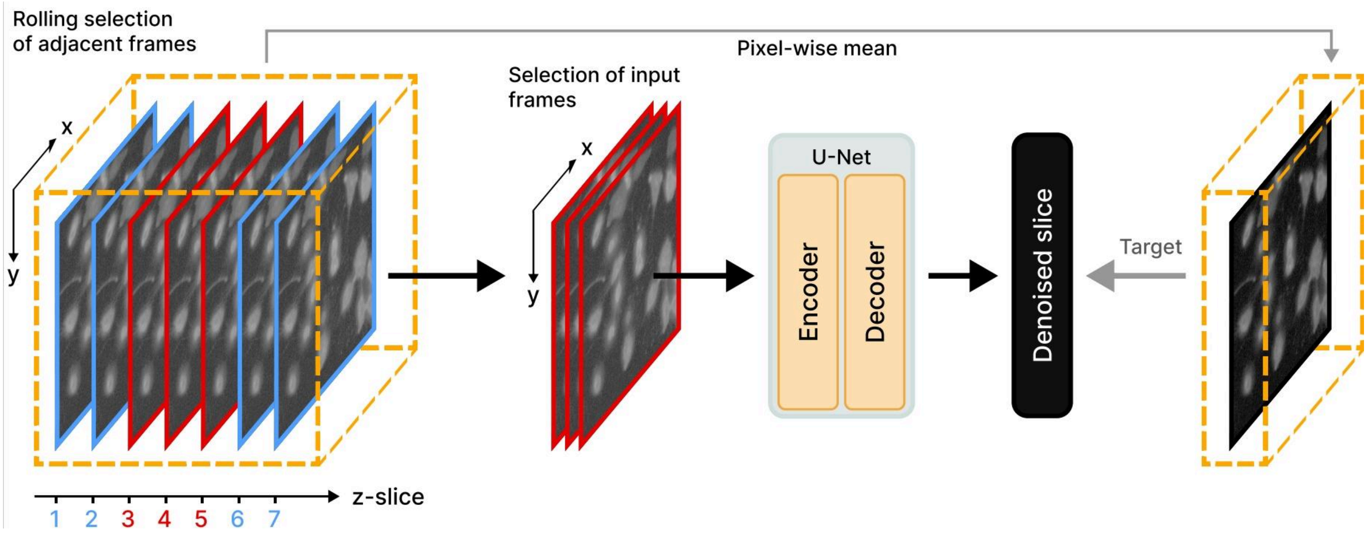

Supplementary Fig. 10 Schematic of denoising multi-frame time-lapse data implemented with U-Net on QIScope.

The U-Net network architecture was applied to the principles of Noise2Noise training. The input to the network are three adjacent frames and the model is trained to predict the central frame. During training, the target is the pixel-wise mean of seven adjacent frames centered around the frame that is being denoised. By passing multiple frames as input, the model is provided with more information about the structures within the frames, which leads to more robust restoration performance.

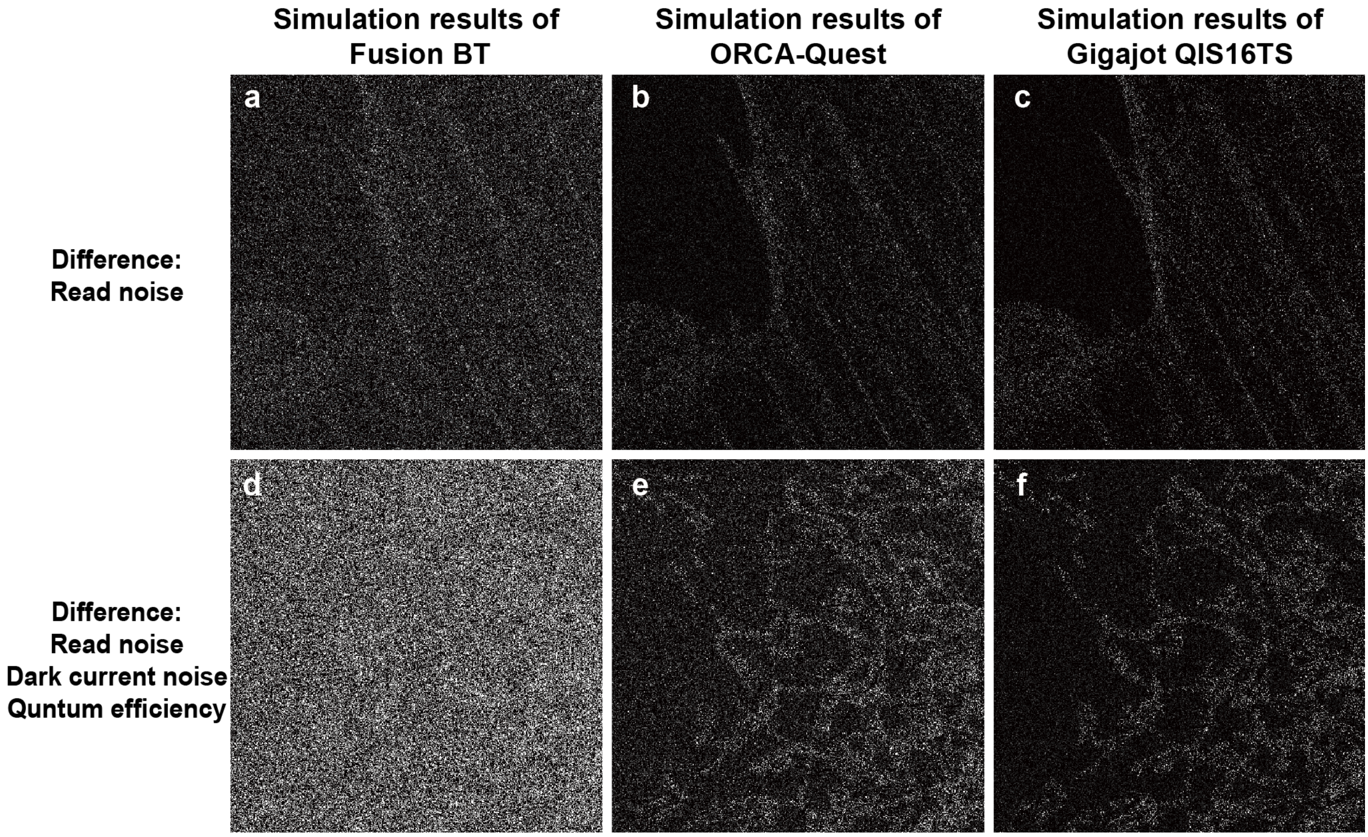

Supplementary Fig. 11 | Simulation of Hamamatsu Fusion BT (sCMOS), ORCA-Quest, and Gigajot QIS16TS using the Hamamatsu Camera Simulation Engine.

a–c, Simulation results of Fusion BT (**a**), ORCA-Quest (**b**), and Gigajot QIS16TS (**c**) with all camera parameters set to be identical except for camera read noise, which reflects company-specified read noise values for each camera. **d–f,** Similar simulations as in **a–c**, except read noise, quantum efficiency, and dark current noise are set to company-specified values, with all other parameters being equal. Representative results are shown from three independent simulations.

Supplementary Table 1 | Comparison of Camera Specifications for the Hamamatsu Fusion BT, Andor iXon 897, Hamamatsu ORCA-Quest qCMOS, and Gigajot QIS16TS

Camera	Hamamatsu Fusion BT sCMOS	Andor iXon 897 EMCCD	ORCA-Quest qCMOS	Gigajot QIS16TS
**Pixel size**	6.5 μm × 6.5 μm	16 μm × 16 μm	4.6 μm × 4.6 μm	**1.1 μm × 1.1 μm**
**Pixel number**	2304 × 2304	512 × 512	4096 × 2304	4096 × 4096
**Chip size**	14.976 mm × 14.976 mm	8.2mm × 8.2 mm	18.841 mm × 10.598 mm	4.5 mm × 4.5 mm
**Chip area**	224.28 mm^2	67.24 mm^2	199.68 mm^2	20.25 mm^2
**Quantum efficiency**	95% @550 nm	>95% @peak	85% @peak	81% @peak
**Read noise**	0.7 electrons rms @Ultra-quiet scan	<1 electrons rms (EM gain >50)	0.27 electrons rms @Ultra-quiet scan	**0.19 electrons rms @Photon Number Resolving**
**Dark current noise**	0.7 e-/sec/pix @ less than -15°C	0.00015 e-/sec/pix @ less than -80°C	0.016 e-/sec/pix @ less than -20°C	**0.002 e-/sec/pix @ less than 10°C**
**Frame rate**	5.4 fps @Ultra-quiet scan	56 fps	5 fps @Ultra-quiet scan	10 fps

Supplementary Table 2 | Specifications for setups built for comparing cameras at the same effective pixel size

	Hamamatsu Fusion BT sCMOS	Andor iXon 897 EMCCD	Gigajot QIS16TS
**Pixel size**	6.5 μm	16 μm	1.1 μm
**Objective lens**	100x oil NA:1.45 (UPLXAPO100XO, Olympus)	100x oil NA:1.45 (UPLXAPO100XO, Olympus)	40X oil NA:1.4 (UPLXAPO40XO, Olympus)
**Tube lens**	80 mm (AC254-080-A-ML, Thorlabs)	200 mm (ACT508-200-A-ML, Thorlabs)	35 mm (AC254-035-A-ML, Thorlabs)
**Magnification**	44.44 X	111.11 X	7.78X
**Effective pixel size**	**0.146 μm**	**0.144 μm**	**0.141 μm**

Supplementary Table 3 | Signal-to-noise ratios obtained for camera comparisons using ND filters and same effective pixel size

Camera		SNR of Hamamatsu Fusion BT	SNR of Andor iXon 897	SNR of Gigajot QIS16TS
ND filter	Fluorescence slide	39.9	86.0	390.182
	Cells with QDs	0.6	3.6	14.171
Same effective pixel size	Fluorescence slide	29.4	82.9	348.850
	Cells with QDs	36.5	101.5	460.111

Supplementary Table 4 | Details of setups built for comparing the EMCCD and QIScope at the same effective pixel size

Camera	Optical setup and EMCCD	QIScope
**Pixel size**	16 μm	1.1 μm
**Objective lens**	40x oil (UPLXAPO40XO, Olympus)	40X oil (UPLXAPO40XO, Olympus)
**Tube lens**	180 mm (AC508-180-A-ML,Thorlabs)	20X (LUCPLFLN20X, Olympus) add two lenses (AC254-045-A-ML and AC254-035-A-ML)
**Magnification**	40X	2.6X
**Effective pixel size**	**0.4 μm**	**0.423 μm**

Supplementary Note

CDNA Sequences for cell line expression

ATG (start codon)

Kozak sequence

ALFA tag

NLuc

Linker (SGGS)

MyrPalm sequence of LCK (mus musculus) (1x myristoylation Gly1, 2 x Palmitoylation Cys2 and Cys4)

CD63

Gamillus or msfGFP

Linker

Flag

Cyto-NLuc

GCCGCCACCATGGTCTTCACACTCGAAGATTTCGTTGGGGACTGGCGACAGACAGCCGGCTACAAC
CTGGACCAAGTCCTTGAACAGGGAGGTGTGTCCAGTTTGTTTCAGAATCTCGGGGTGTCCGTAACT
CCGATCCAAAGGATTGTCCTGAGCGGTGAAAATGGGCTGAAGATCGACATCCATGTCATCATCCCGT
ATGAAGGTCTGAGCGGCGACCAAATGGGCCAGATCGAAAAAATTTTTAAGGTGGTGTACCCTGTGGA
TGATCATCACTTTAAGGTGATCCTGCACTATGGCACACTGGTAATCGACGGGGTTACGCCGAACATGA
TCGACTATTTCGGACGGCCGTATGAAGGCATCGCCGTGTTCGACGGCAAAAAGATCACTGTAACAGG
GACCCTGTGGAACGGCAACAAAATTATCGACGAGCGCCTGATCAACCCCGACGGCTCCCTGCTGTT
CCGAGTAACCATCAACGGAGTGACCGGCTGGCGGCTGTGCGAACGCATTCTGGCTCCCAGCCGGC
TGGAGGAAGAGCTGCGGCGCCGACTGACAGAATGA

MyrPalm(LCK)-NLuc

GCCGCCACCATGGGCTGTGTCTGCAGCTCAAACCCCGAGAACAACAACAACAGTGGAGGGGGCAG
TGTCTTCACACTCGAAGATTTCGTTGGGGACTGGCGACAGACAGCCGGCTACAACCTGGACCAAGT
CCTTGAACAGGGAGGTGTGTCCAGTTTGTTTCAGAATCTCGGGGTGTCCGTAACTCCGATCCAAAG
GATTGTCCTGAGCGGTGAAAATGGGCTGAAGATCGACATCCATGTCATCATCCCGTATGAAGGTCTG
AGCGGCGACCAAATGGGCCAGATCGAAAAAATTTTTAAGGTGGTGTACCCTGTGGATGATCATCACT
TTAAGGTGATCCTGCACTATGGCACACTGGTAATCGACGGGGTTACGCCGAACATGATCGACTATTTC
GGACGGCCGTATGAAGGCATCGCCGTGTTCGACGGCAAAAAGATCACTGTAACAGGGACCCTGTGG
AACGGCAACAAAATTATCGACGAGCGCCTGATCAACCCCGACGGCTCCCTGCTGTTCCGAGTAACC
ATCAACGGAGTGACCGGCTGGCGGCTGTGCGAACGCATTCTGGCTCCCAGCCGGCTGGAGGAAGA
GCTGCGGCGCCGACTGACAGAATGA

NLuc-CD63 (homo sapiens)

GCCGCCACCATGCCCAGCCGGCTGGAGGAAGAGCTGCGGCGCCGACTGACAGAACCTGTCTTCAC
ACTCGAAGATTTCGTTGGGGACTGGCGACAGACAGCCGGCTACAACCTGGACCAAGTCCTTGAACA
GGGAGGTGTGTCCAGTTTGTTTCAGAATCTCGGGGTGTCCGTAACTCCGATCCAAAGGATTGTCCTG
AGCGGTGAAAATGGGCTGAAGATCGACATCCATGTCATCATCCCGTATGAAGGTCTGAGCGGCGAC
CAAATGGGCCAGATCGAAAAAATTTTTAAGGTGGTGTACCCTGTGGATGATCATCACTTTAAGGTGAT
CCTGCACTATGGCACACTGGTAATCGACGGGGTTACGCCGAACATGATCGACTATTTCGGACGGCCG
TATGAAGGCATCGCCGTGTTCGACGGCAAAAAGATCACTGTAACAGGGACCCTGTGGAACGGCAAC
AAAATTATCGACGAGCGCCTGATCAACCCCGACGGCTCCCTGCTGTTCCGAGTAACCATCAACGGA
GTGACCGGCTGGCGGCTGTGCGAACGCATTCTGGCGCTCGAGCCTATGGCGGTGGAAGGAGGAAT
GAAATGTGTGAAGTTCTTGCTCTACGTCCTCCTGCTGGCCTTTTGCGCCTGTGCAGTGGGACTGATT
GCCGTGGGTGTCGGGGCACAGCTTGTCCTGAGTCAGACCATAATCCAGGGGGCTACCCCTGGCTC
TCTGTTGCCAGTGGTCATCATCGCAGTGGGTGTCTTCCTCTTCCTGGTGGCTTTTGTGGGCTGCTGC
GGGGCCTGCAAGGAGAACTATTGTCTTATGATCACGTTTGCCATCTTTCTGTCTCTTATCATGTTGGT
GGAGGTGGCCGCAGCCATTGCTGGCTATGTGTTTAGAGATAAGGTGATGTCAGAGTTTAATAACAACT
TCCGGCAGCAGATGGAGAATTACCCGAAAAATAACCACACTGCTTCGATCCTGGACAGGATGCAGG
CAGATTTTAAGTGCTGTGGGGCTGCTAACTACACAGATTGGGAGAAAATCCCTTCCATGTCGAAGAA
CCGAGTCCCCGACTCCTGCTGCATTAATGTTACTGTGGGCTGTGGGATTAATTTCAACGAGAAGGCG

ATCCATAAGGAGGGCTGTGTGGAGAAGATTGGGGGCTGGCTGAGGAAAAATGTGCTGGTGGTAGCT
GCAGCAGCCCTTGGAATTGCTTTTGTCGAGGTTTTGGGAATTGTCTTTGCCTGCTGCCTCGTGAAGA
GTATCAGAAGTGGCTACGAGGTGATGTAG

Gamillus-Linker-CD63

GCCGCCACCATGGTGTCCAAGGGAGAAGAAGCGAGTGGTCGGGCTCTGTTTCAGTACCCTATGACG
AGCAAAATAGAGCTTAATGGGGAGATTAACGGAAAAAAGTTTAAGGTTGCCGGGGAGGGGTTTACCC
CGTCCTCTGGGCGATTCAACATGCATGCTTACTGCACCACGGGCGATCTCCCTATGTCTTGGGTTGT
CATCGCCTCACCCCTGCAGTATGGCTTTCACATGTTTGCACATTACCCGGAGGACATTACACACTTTT
TCCAGGAGTGCTTCCCCGGTTCATACACTCTCGATCGGACTCTGAGGATGGAAGGAGACGGAACCC
TGACCACTCACCATGAGTATAGCCTTGAGGACGGATGTGTCACCTCAAAAACAACACTTAATGCTTCA
GGGTTCGATCCGAAAGGAGCAACCATGACCAAGTCCTTCGTAAAGCAGCTCCCCAACGAAGTGAAA
ATCACGCCGCACGGACCGAATGGGATTAGATTGACAAGTACGGTACTCTATCTTAAGGAAGACGGCA
CTATACAGATAGGGACGCAAGATTGTATTGTTACCCCCGTGGGCGGGAGAAAAGTGACACAACCCAA
AGCACATTTCCTCCACACTCAGATTATCCAGAAAAAGGATCCCAACGACACCCGAGATCACATCGTAC
AAACTGAACTCGCGGTTGCTGGTAACCTCTGGCACGGAATGGACGAACTTTACAAGCCCAGCCGGC
TGGAGGAAGAGCTGCGGCGCCGACTGACAGAACCTGCGGTGGAAGGAGGAATGAAATGTGTGAAG
TTCTTGCTCTACGTCCTCCTGCTGGCCTTTTGCGCCTGTGCAGTGGGACTGATTGCCGTGGGTGTC
GGGGCACAGCTTGTCCTGAGTCAGACCATAATCCAGGGGGCTACCCCTGGCTCTCTGTTGCCAGTG
GTCATCATCGCAGTGGGTGTCTTCCTCTTCCTGGTGGCTTTTGTGGGCTGCTGCGGGGCCTGCAAG
GAGAACTATTGTCTTATGATCACGTTTGCCATCTTTCTGTCTCTTATCATGTTGGTGGAGGTGGCCGC
AGCCATTGCTGGCTATGTGTTTAGAGATAAGGTGATGTCAGAGTTTAATAACAACTTCCGGCAGCAGA
TGGAGAATTACCCGAAAAATAACCACACTGCTTCGATCCTGGACAGGATGCAGGCAGATTTTAAGTG
CTGTGGGGCTGCTAACTACACAGATTGGGAGAAAATCCCTTCCATGTCGAAGAACCGAGTCCCCGA
CTCCTGCTGCATTAATGTTACTGTGGGCTGTGGGATTAATTTCAACGAGAAGGCGATCCATAAGGAGG
GCTGTGTGGAGAAGATTGGGGGCTGGCTGAGGAAAAATGTGCTGGTGGTAGCTGCAGCAGCCCTT
GGAATTGCTTTTGTCGAGGTTTTGGGAATTGTCTTTGCCTGCTGCCTCGTGAAGAGTATCAGAAGTG
GCTACGAGGTGATGTAG

NLuc-Linker-Gamillus-Flag

ATGGTCTTCACACTCGAAGATTTCGTTGGGGACTGGCGACAGACAGCCGGCTACAACCTGGACCAA
GTCCTTGAACAGGGAGGTGTGTCCAGTTTGTTTCAGAATCTCGGGGTGTCCGTAACTCCGATCCAAA
GGATTGTCCTGAGCGGTGAAAATGGGCTGAAGATCGACATCCATGTCATCATCCCGTATGAAGGTCT
GAGCGGCGACCAAATGGGCCAGATCGAAAAAATTTTTAAGGTGGTGTACCCTGTGGATGATCATCAC
TTTAAGGTGATCCTGCACTATGGCACACTGGTAATCGACGGGGTTACGCCGAACATGATCGACTATTT
CGGACGGCCGTATGAAGGCATCGCCGTGTTCGACGGCAAAAAGATCACTGTAACAGGGACCCTGTG
GAACGGCAACAAAATTATCGACGAGCGCCTGATCAACCCCGACGGCTCCCTGCTGTTCCGAGTAAC
CATCAACGGAGTGACCGGCTGGCGGCTGTGCGAACGCATTCTGGCTGGTGGAGGTGGATCTGGTG
GAGGTGGATCAGTGTCCAAGGGAGAAGAAGCGAGTGGTCGGGCTCTGTTTCAGTACCCTATGACGA
GCAAAATAGAGCTTAATGGGGAGATTAACGGAAAAAAGTTTAAGGTTGCCGGGGAGGGGTTTACCCC
GTCCTCTGGGCGATTCAACATGCATGCTTACTGCACCACGGGCGATCTCCCTATGTCTTGGGTTGTC
ATCGCCTCACCCCTGCAGTATGGCTTTCACATGTTTGCACATTACCCGGAGGACATTACACACTTTTT
CCAGGAGTGCTTCCCCGGTTCATACACTCTCGATCGGACTCTGAGGATGGAAGGAGACGGAACCCT
GACCACTCACCATGAGTATAGCCTTGAGGACGGATGTGTCACCTCAAAAACAACACTTAATGCTTCA
GGGTTCGATCCGAAAGGAGCAACCATGACCAAGTCCTTCGTAAAGCAGCTCCCCAACGAAGTGAAA
ATCACGCCGCACGGACCGAATGGGATTAGATTGACAAGTACGGTACTCTATCTTAAGGAAGACGGCA
CTATACAGATAGGGACGCAAGATTGTATTGTTACCCCCGTGGGCGGGAGAAAAGTGACACAACCCAA
AGCACATTTCCTCCACACTCAGATTATCCAGAAAAAGGATCCCAACGACACCCGAGATCACATCGTAC
AAACTGAACTCGCGGTTGCTGGTAACCTCTGGCACGGAATGGACGAACTTTACAAGGACTACAAAGA
CGATGATGACAAGTAA

TRE3g-driven NLuc-msfGFP (Tet-ON 3g transactivator)

GCCGCCACCATGGTCTTTACACTGGAAGATTTCGTCGGCGACTGGCGGCAGACAGCTGGCTACAAT
CTGGACCAGGTGCTGGAACAAGGCGGCGTGTCCTCTCTGTTTCAAAACCTGGGAGTGTCTGTGACC
CCTATCCAGAGAATCGTGCTGAGCGGCGAGAACGGCCTGAAGATCGACATCCACGTGATCATCCCT
TACGAGGGCCTGTCCGGCGATCAGATGGGACAGATCGAGAAGATCTTTAAGGTGGTGTACCCCGTG
GACGACCACCACTTCAAAGTGATCCTGCACTACGGCACCCTGGTCATCGATGGCGTGACCCCAAAC
ATGATCGACTACTTCGGCAGACCCTACGAGGGAATCGCCGTGTTCGACGGCAAGAAAATCACCGTG
ACCGGCACACTGTGGAACGGCAACAAGATCATCGACGAGAGACTGATCAACCCCGACGGCAGCCT
GCTGTTCAGAGTGACAATCAACGGCGTGACAGGCTGGCGGCTGTGCGAAAGAATCCTTGCTGGAG
GATCCGGCGGCTCTGTGAGCAAGGGCGAAGAACTGTTCACAGGCGTGGTGCCTATCCTGGTGGAA
CTGGATGGGGATGTGAACGGCCACAAGTTCAGCGTCAGAGGCGAAGGCGAAGGGGATGCCACAAA
CGGCAAGCTGACCCTGAAGTTCATCTGCACCACCGGAAAGCTGCCCGTGCCTTGGCCTACACTGGT
CACAACACTGACCTACGGCGTGCAGTGCTTCAGCAGATACCCCGACCATATGAAGCAGCACGACTT
CTTCAAGAGCGCCATGCCTGAGGGCTACGTGCAAGAGAGAACCATCAGCTTCAAGGACGACGGCA
CCTACAAGACCAGAGCCGAAGTGAAGTTCGAGGGCGACACCCTGGTCAACAGAATCGAGCTGAAG
GGCATCGACTTCAAAGAGGACGGCAACATCCTGGGCCACAAACTTGAGTACAACTTCAACAGCCAC
AACGTCTACATCACCGCCGACAAGCAGAAGAACGGCATCAAGGCCAACTTCAAGATCAGGCACAAC
GTGGAAGATGGCAGCGTGCAGCTGGCCGATCACTACCAGCAGAACACACCTATCGGCGACGGACC
TGTGCTGCTGCCTGATAACCACTACCTGAGCACCCAGAGCAAGCTGAGCAAGGACCCCAACGAGAA
GAGGGACCACATGGTGCTGCACGAGTTCGTGACAGCCGCTGGCATCACACTCGGCATGGACGAGC
TGTATAAGTGA
