# Supplementary material for: A telescopic microscope equipped with a quanta image sensor for live-cell bioluminescence imaging
Source: Nat Methods. 2025 May 29;22(6):1321–30. doi: 10.1038/s41592-025-02694-3 (PMC12165864; doi:10.1038/s41592-025-02694-3)
Supplement: Supplementary file 2 — Reporting Summary [file 41592_2025_2694_MOESM2_ESM.pdf]

nature portfolio

Corresponding author(s): Jian Cui

Last updated by author(s): Jan 23, 2025

Reporting Summary

Nature Portfolio wishes to improve the reproducibility of the work that we publish. This form provides structure for consistency and transparency in reporting. For further information on Nature Portfolio policies, see our Editorial Policies and the Editorial Policy Checklist.

Statistics

For all statistical analyses, confirm that the following items are present in the figure legend, table legend, main text, or Methods section.

n/a	Confirmed	
☐	☒	The exact sample size (*n*) for each experimental group/condition, given as a discrete number and unit of measurement
☒	☐	A statement on whether measurements were taken from distinct samples or whether the same sample was measured repeatedly
☒	☐	The statistical test(s) used AND whether they are one- or two-sided<br>*Only common tests should be described solely by name; describe more complex techniques in the Methods section.*
☒	☐	A description of all covariates tested
☒	☐	A description of any assumptions or corrections, such as tests of normality and adjustment for multiple comparisons
☒	☐	A full description of the statistical parameters including central tendency (e.g. means) or other basic estimates (e.g. regression coefficient) AND variation (e.g. standard deviation) or associated estimates of uncertainty (e.g. confidence intervals)
☒	☐	For null hypothesis testing, the test statistic (e.g. F, t, r) with confidence intervals, effect sizes, degrees of freedom and P value noted<br>*Give P values as exact values whenever suitable.*
☒	☐	For Bayesian analysis, information on the choice of priors and Markov chain Monte Carlo settings
☒	☐	For hierarchical and complex designs, identification of the appropriate level for tests and full reporting of outcomes
☒	☐	Estimates of effect sizes (e.g. Cohen's *d*, Pearson's *r*), indicating how they were calculated

Our web collection on statistics for biologists contains articles on many of the points above.

Software and code

Policy information about availability of computer code

Data collection	Images were collected using the Hamamatsu Hokawo v3.0 (sCMOS), the Andor Solis v4.31.30023.0 (EMCCD), and the Gigajot Nectarine v1.5 (QIS).
Data analysis	Images were processed using ImageJ 1.53c and simple code written in Python 3.8.5. Videos were processed using ImageJ 1.53c and Adobe Premiere Pro 2023. Denoising was performed using Noise2Noise and Noise2Info as described in the Methods. Code used in the manuscript can be found at https://github.com/RuyuMa/A-Telescopic-Microscope-Equipped-with-a-Quanta-Image-Sensor-for-Live-Cell-Bioluminescence-Imaging

For manuscripts utilizing custom algorithms or software that are central to the research but not yet described in published literature, software must be made available to editors and reviewers. We strongly encourage code deposition in a community repository (e.g. GitHub). See the Nature Portfolio guidelines for submitting code & software for further information.

Data

Policy information about availability of data

All manuscripts must include a data availability statement. This statement should provide the following information, where applicable:

- Accession codes, unique identifiers, or web links for publicly available datasets
- A description of any restrictions on data availability
- For clinical datasets or third party data, please ensure that the statement adheres to our policy

Raw data for main text figures and extended data figures is publicly available at https://doi.org/10.5281/zenodo.14726231

Research involving human participants, their data, or biological material

Policy information about studies with human participants or human data. See also policy information about sex, gender (identity/presentation), and sexual orientation and race, ethnicity and racism.

Reporting on sex and gender	No research on human participants.
Reporting on race, ethnicity, or other socially relevant groupings	No research on human participants.
Population characteristics	No research on human participants.
Recruitment	No research on human participants.
Ethics oversight	No research on human participants.

Note that full information on the approval of the study protocol must also be provided in the manuscript.

Field-specific reporting

Please select the one below that is the best fit for your research. If you are not sure, read the appropriate sections before making your selection.

☒ Life sciences ☐ Behavioural & social sciences ☐ Ecological, evolutionary & environmental sciences

For a reference copy of the document with all sections, see nature.com/documents/nr-reporting-summary-flat.pdf

Life sciences study design

All studies must disclose on these points even when the disclosure is negative.

Sample size	Sample sizes were determined to ensure reproducibility in order to draw conclusions about the capabilities of this technology.
Data exclusions	No data was "excluded" per se. Representative images were selected to make the appropriate points.
Replication	The number of reproduced measurements are included in the Figure captions.
Randomization	For measurements of cells, random cells were imaged. No additional randomization was performed nor necessary to demonstrate the capabilities of the microscope.
Blinding	No blinding could be performed as the same researcher often prepared samples, collected images, and analyzed images.

Reporting for specific materials, systems and methods

We require information from authors about some types of materials, experimental systems and methods used in many studies. Here, indicate whether each material, system or method listed is relevant to your study. If you are not sure if a list item applies to your research, read the appropriate section before selecting a response.

Materials & experimental systems

n/a	Involved in the study
☐	☒ Antibodies
☐	☒ Eukaryotic cell lines
☒	☐ Palaeontology and archaeology
☒	☐ Animals and other organisms
☒	☐ Clinical data
☒	☐ Dual use research of concern
☒	☐ Plants

Methods

n/a	Involved in the study
☒	☐ ChIP-seq
☒	☐ Flow cytometry
☒	☐ MRI-based neuroimaging

Antibodies

Antibodies used

1) Smooth muscle actin Polyclonal antibody, Proteintech 14395-I-AP.
2) Secondary Antibody conjugated to Qdot 585, ThermoFisher Q-11411MP. Specificity not necessary for our measurements.
3) Recombinant anti-ALFA single-domain antibody fused to a Guinea Pig IgG Fc domain, NanoTag Biotechnologies N1584.
4) Goat Anti-Guinea pig IgG H&L (HRP), Abcam ab6908.

Validation

1) Proteintech 14395-I-AP: Manufacturer states it is knockdown/knockout validated. Multiple western blots provided.
2) ThermoFisher Q-11411MP: Manufacturer supplies data showing no non-specific immunofluorescence signal with secondary antibody alone in HeLa cells.
3) NanoTag Biotechnologies N1584: Validation data published in Götzke et al. Nat Commun 10, 4403 (2019). Revalidated in Extended Data Fig. 4 by western blot.
4) Abcam ab6908: No validation data provided by the manufacturer. Validated in Extended Data Fig. 4 by western blot.

Eukaryotic cell lines

Policy information about cell lines and Sex and Gender in Research

Cell line source(s)

The cell line carrying EXSISERS was created as described in Truong et al. 2021 (DOI 10.1038/s41556-021-00678-x), who are also co-authors on this manuscript.
The EV cell lines, PINK1 cell line, Gamillus-NLuc cell line, and NLuc-msfGFP cell line were obtained as described in the Methods.

Authentication

EXSISERS: Genetic authentication was performed in the original manuscript by Truong et al. 2021 (DOI 10.1038/s41556-021-00678-x)
EV cell lines: Protein expression was verified by western blot and size exclusion chromatography as shown in Supplementary Fig. 4 and 5.
PINK1, Gamillus-NLuc, and NLuc-msfGFP cell lines were verified by bioluminescence and fluorescence imaging as in Fig. 5 and Supplementary Fig. 9.

Mycoplasma contamination

EXSISERS, PINK1, and NLuc-msfGFP cells were regularly checked via Hoechst 3334, which visualizes extranuclear speckles in the case of contamination. EV and Gamillus-NLuc cell lines were regularly tested for mycoplasma infection by PCR. No evidence of contamination was observed.

Commonly misidentified lines
(See ICLAC register)

N/A

Plants

Seed stocks

N/A

Novel plant genotypes

N/A

Authentication

N/A
